# Design, synthesis and biological evaluation of a series of CNS penetrant HDAC inhibitors structurally derived from amyloid-*β* probes

**DOI:** 10.1038/s41598-019-49784-9

**Published:** 2019-09-12

**Authors:** Myeong A Choi, Sun You Park, Hye Yun Chae, Yoojin Song, Chiranjeev Sharma, Young Ho Seo

**Affiliations:** College of Pharmacy, Keimyung University, Daegu, 42601 South Korea

**Keywords:** Drug discovery and development, Structure-based drug design

## Abstract

To develop novel CNS penetrant HDAC inhibitors, a new series of HDAC inhibitors having benzoheterocycle were designed, synthesized, and biologically evaluated. Among the synthesized compounds, benzothiazole derivative **9b** exhibited a remarkable anti-proliferative activity (GI_50_ = 2.01 μM) against SH-SY5Y cancer cell line in a dose and time-dependent manner, better than the reference drug SAHA (GI_50_ = 2.90 μM). Moreover, compound **9b** effectively promoted the accumulation of acetylated Histone H3 and *α*-tubulin through inhibition of HDAC1 and HDAC6 enzymes, respectively. HDAC enzyme assay also confirmed that compound **9b** efficiently inhibited HDAC1 and HDAC6 isoforms with IC_50_ values of 84.9 nM and 95.9 nM. Furthermore, compound **9b** inhibited colony formation capacity of SH-SY5Y cells, which is considered a hallmark of cell carcinogenesis and metastatic potential. The theoretical prediction, *in vitro* PAMPA-BBB assay, and *in vivo* brain pharmacokinetic studies confirmed that compound **9b** had much higher BBB permeability than SAHA. *In silico* docking study demonstrated that compound **9b** fitted in the substrate binding pocket of HDAC1 and HDAC6. Taken together, compound **9b** provided a novel scaffold for developing CNS penetrant HDAC inhibitors and therapeutic potential for CNS-related diseases.

## Introduction

Histone deacetylase (HDACs) and histone acetyltransferases (HATs) control the dynamic status of histone acetylation, which plays an important role in the regulation of gene expression. In general, the hyper-acetylation of histones is associated with transcriptional gene activation. Conversely, the hypo-acetylation of histones is correlated with transcriptional gene repression. HDACs remove the acetyl groups from hyper-acetylated histones, resulting in a closed chromatin configuration that blocks the access of the transcription machinery to DNA, and consequently suppress gene expression^1^. In contrast, HATs acetylate the lysine residues of histones, opposing the effect of HDACs and that leads to a relaxed chromatin structure, which enhances gene transcription. In addition to their roles in the transcriptional gene regulation, HDACs are also involved in the acetylation of various non-histone proteins such as Hsp90, *α*-tubulin, p53, Foxp3, E2F1, and NF-*κ*B^2,3^.

HDACs are composed of eleven zinc-dependent enzymes, which are further divided into four distinct classes, including class I (HDACs 1, 2, 3, and 8), class IIa (HDACs 4, 5, 7, and 9), class IIb (HDACs 6 and 10), and class IV (HDAC11)^4^. NAD^+^-dependent HDAC enzymes, known as sirtuins belong to class III (sirtuins 1–7). Over the past decade, HDACs have emerged as promising targets with a broad range of potential indications, such as cancers and CNS disorders^5–7^.

**Figure 1:**
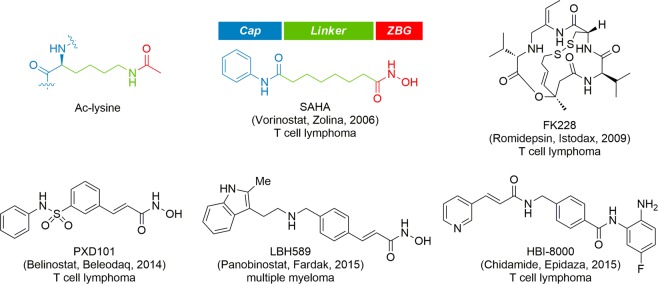
Structures of clinically approved HDAC inhibitors.

HDACs have been primarily investigated as anticancer targets and accordingly numerous HDAC inhibitors are currently at various stages of pre-clinical and clinical trials for the treatment of cancers^8–15^. To date, five HDAC inhibitors are approved globally. Four HDAC inhibitors including SAHA, FK228, FXD101, and LBH589 are clinically approved by the US FDA for the treatment of cutaneous T-cell lymphoma and multiple myeloma, and a HDAC inhibitor, HBI-8000 is approved by the Chinese FDA for the treatment of cutaneous T-cell lymphoma (Fig. 1)^16–20^.

Although most HDAC inhibitors have been developed for the hematological malignancies, an increasing number of HDAC inhibitors are investigated for the treatment of central nervous system (CNS) diseases such as brain cancers, Alzheimer’s disease, depression, and drug addiction^6,21^. It has been reported that a number of HDAC inhibitors such as SAHA, MS-275, tubastatin A, and valproic acid have low brain uptake due to poor blood-brain barrier (BBB) permeability, highlighting their limitation as clinical applications for CNS diseases^22–27^. Only a handful of CNS-penetrant HDAC inhibitors have recently been reported for their therapeutic potential in CNS disorders^28–32^. Despite the challenges and difficulties in the drug discovery of CNS therapeutics, the potential therapeutic benefits of HDAC inhibitors in CNS diseases prompted us to develop CNS penetrant HDAC inhibitors structurally distinct from the previous reported CNS-penetrant HDAC inhibitors.

## Results and Discussion

### Chemistry

**Figure 2:**
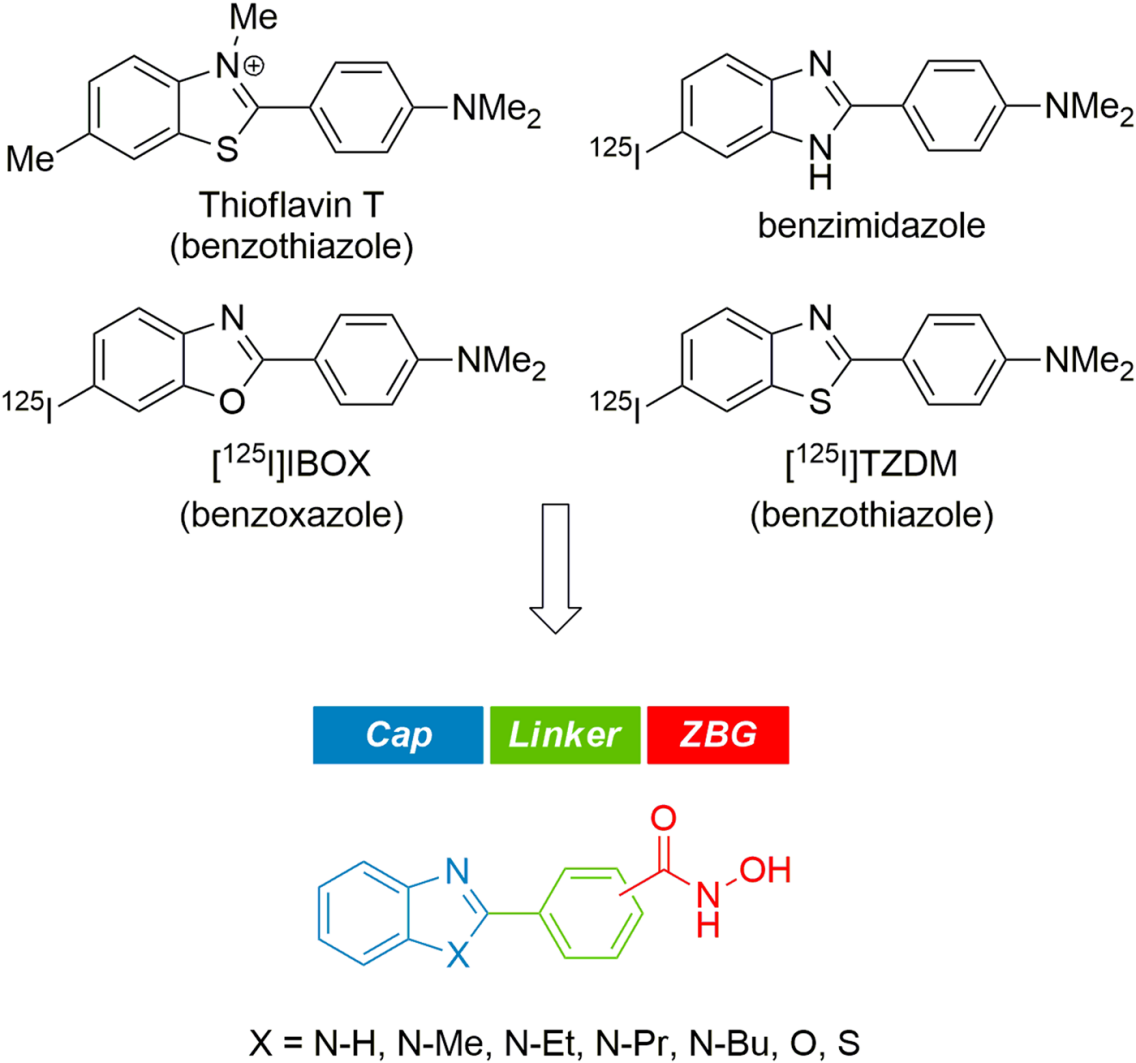
Design of HDAC inhibitors derived from structures of amyloid-*β* probes.

During the past decade, substantial efforts have been undertaken to develop amyloid-*β* probes with a high brain uptake, resulting in a variety of radiolabeled molecular probes for *in vivo* amyloid-*β* imaging (Fig. 2)^33–35^. Among the scaffolds derived from these molecular probes, thioflavin-T analogues such as benzothiazole, benzoxazole, and benzimidazole display not only excellent binding affinity to amyloid-*β* aggregates but also high uptake into the brain. Besides, these benzoheterocycles have also attracted attention in oncology due to their diverse biological activities and applications in cancer treatment^36,37^. On the basis of their excellent BBB penetrating property and common pharmacophores in oncology, we assumed that these benzoheterocycle scaffolds might be a viable starting point for the development of CNS penetrant HDAC inhibitors. Therefore, we set out to design HDAC inhibitors structurally derived from amyloid-*β* probes. HDAC inhibitors share a common structural features including capping group, a linker group, and a zinc-binding group (ZBG), which have been widely employed in the design of HDAC inhibitors. Benefiting from this pharmacophore model, we designed a series of HDAC inhibitors bearing a benzoheterocyle cap, a phenyl linker, and hydroxamic acid ZBG, as shown in Fig. 2.

**Figure 3:**
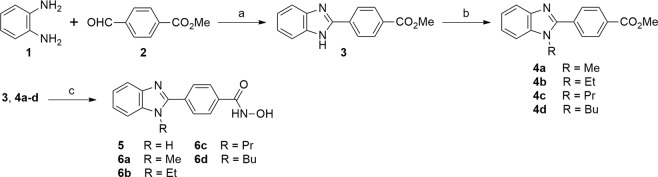
Synthesis of compounds **5** and **6a–d**. Reagents and conditions: (**a**) 10% H_2_O in DMF, 80 °C, 36 h, 88%; (**b**) NaH, alkyl iodide, DMF, rt, 10 h, 45–70%; (**c**) NH_2_OH, KOH, MeOH, 0 °C, 3 h, 36–51%.

We first commenced the synthesis of compounds **5** and **6a–d**, illustrated in Fig. 3. Compound **3** was synthesized following the previously reported procedure with a slight modification^38^. Briefly, reaction of *ortho*-phenylenediamine (**1**) with aldehyde **2** in aqueous DMF provided a key intermediate **3** via aerobic oxidation in 88% yield. After that, compound **3** was treated with sodium hydride in DMF for 2 h, followed by the addition of various alkyl iodide to provide compounds **4a–d** in 45–70% yield. Finally, subsequent treatment of **3** or **4a–d** with NH_2_OH and KOH in methanol provided compounds **5** or **6a–d** in 32–35% yield.

**Figure 4:**
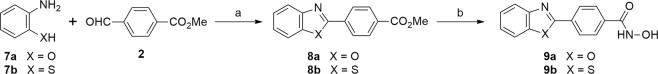
Synthesis of compounds **9a-b**. Reagents and conditions: (**a**) I_2_, DCM, rt, 3 h, 32% for **8a**, 35% for **8b**; (**b**) NH_2_OH, KOH, MeOH, 0 °C, 3 h, 45% for **9a**, 42% for **9b**.

We next pursued the synthesis of **9a-b** depicted in Fig. 4. Compounds **8a-b** were prepared by iodine-mediated cyclization of the corresponding starting materials, 2-aminophenol (**7a**) or 2-aminothiophenol (**7b**) with aldehyde **2** in 32–35% yield^39^. Subsequent treatment of ester **8a-b** with NH_2_OH and KOH in methanol successfully furnish compounds **9a-b** in 42–45% yield.

**Figure 5:**
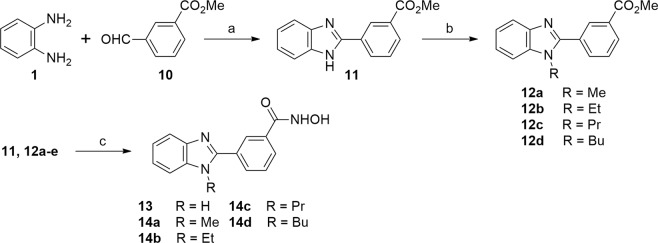
Synthesis of compounds **13** and **14a-e**. Reagents and conditions: (**a**) 10% H_2_O in DMF, 80 °C, 48 h, 86%; (**b**) NaH, alkyl iodide, DMF, rt, 10 h, 11–91%; (**c**) NH_2_OH, KOH, MeOH, 0 °C, 3 h, 33–55%.

To explore the biological activity of *meta*-regioisomers, we further synthezised compounds **13** and **14a–d** depicted in Fig. 5. Similarly, compound **11** was prepared from *ortho*-phenylenediamine (**1**) and aldehyde **10** in aqueous DMF in 86% yield. After that, compound **11** was treated with sodium hydride in DMF for 2 h and then alkylated with the corresponding alkyl iodide to give compounds **12a–d** in 11–91% yield. Synthesis of compounds **13** and **14a–d** was finally achieved through the reaction of compound **11** or **12a–d** with NH_2_OH and KOH in methanol in 33–55% yield.

**Figure 6:**
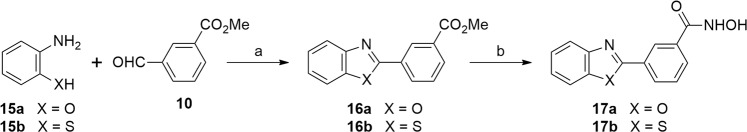
Synthesis of compounds **17a-b**. Reagents and conditions: (**a**) NaCN, molecular sieves, DMF, 60 °C, 48 h, 67% for **16a**, 44% for **16b**. (**b**) NH_2_OH, KOH, MeOH, 0 °C, 3 h, 50% for **17a**, 34% for **17b**.

The first attempt to construct compounds **16a-b** via iodine-mediated cyclization of **15a-b** with aldehyde **10** did not afford the desired product, unlike the reactions in Fig. 4. Therefore, we carried out sodium cyanide-catalyzed cyclization of compounds **15a-b** with aldehyde **10** in DMF, which successfully afforded compounds **16a-b** in 44–67% yield (Fig. 6)^40^. Finally, we accomplished the conversion of compounds **16a-b** to **17a-b** with NH_2_OH and KOH in methanol in 34–50% yield.

### *In vitro* anti-proliferation assays

Upon completion of synthesis, we next investigated their anti-proliferative effect on human neuroblastoma cell line SH-SY5Y, which is an *in vitro* model of human malignant metastatic neuroblastoma. As shown in Table 1, analogue **9b** exerted the most potent anti-proliferative activity with IC_50_ value of 2.01 μM against SH-SY5Y cell line, in that the reference drug SAHA also furnished good anti-proliferative activity with IC_50_ value of 2.90 μM against SH-SY5Y cell line. *Para*-substituted benzimidazole analogues **5** and **6a–d** exhibited relatively poor anti-proliferative activities (IC_50_ = 38.1–60 μM), compared with benzothiazole analogue **9b**. Interestingly, butyl substituent on nitrogen atom of benzimidazole group (**6d**) resulted in approximately a 2-fold increase in potency (26.8 μM) from non-substituted analogue **5** (60 μM). Benzoxazole analogue **9a**, which has oxygen atom replacing sulfur atom of the most potent analogue **9b**, furnished 12-fold less potent anti-proliferative activity (IC_50_ = 25.7 μM) than compound **9b**. It is probably because the hydrophobicity of sulfur atom plays a critical role in the binding to HDAC enzymes and cell permeability. We additionally confirmed that compound **9b** more efficiently promoted the acetylation of Histone H3 and *α*-tubulin than compound **9a** *via* the inhibition of HDAC1 and HDAC6, which is a good agreement with their anti-proliferative activities (Fig. S1). In contrast, the similar structural modification of compound **14a** and **14b** did not have a significant effect on GI_50_ values of compound **14a** and **14b**. Ethyl, propyl and butyl substituents on nitrogen atom of *meta*-substituted benzimidazole moiety, including analogues **14b-d** also increased the cellular anti-proliferative activities against SH-SY5Y cell line, compared with methyl substituted analogue **13** and non-substituted analogue **14a**. However, all *meta*-substituted analogues (**13**, **14a–d**, and **17a-b**) exerted relatively poor or modest anti-proliferative activities, compared with *para*-substituted benzothiazole **9b**. Accordingly, we chose compound **9b** for further biological evaluation.

**Table 1:** Anti-proliferative effect of compounds on SH-SY5Y cells^*a*^.

Compound	SH-SY5Y (GI_50_; μM)	Compound	SH-SY5Y (GI_50_; μM)
**5**	60 ± 1.5	**13**	>100
**6a**	58.3 ± 10.8	**14a**	>100
**6b**	38.1 ± 0.2	**14b**	28.2 ± 0.75
**6c**	54.7 ± 0.95	**14c**	34.6 ± 0.6
**6d**	26.8 ± 1.35	**14d**	20.7 ± 1.0
**9a**	25.7 ± 0.05	**17a**	17.5 ± 0.35
**9b**	2.01 ± 0.33	**17b**	21.4 ± 1.65
SAHA	2.90 ± 0.28		

^*a*^Cytotoxicity on SH-SY5Y neuroblastoma cell line (72 h). Data are expressed as the mean ± SD (n = 4).

### Calculation of physiochemical properties

**Figure 7:**
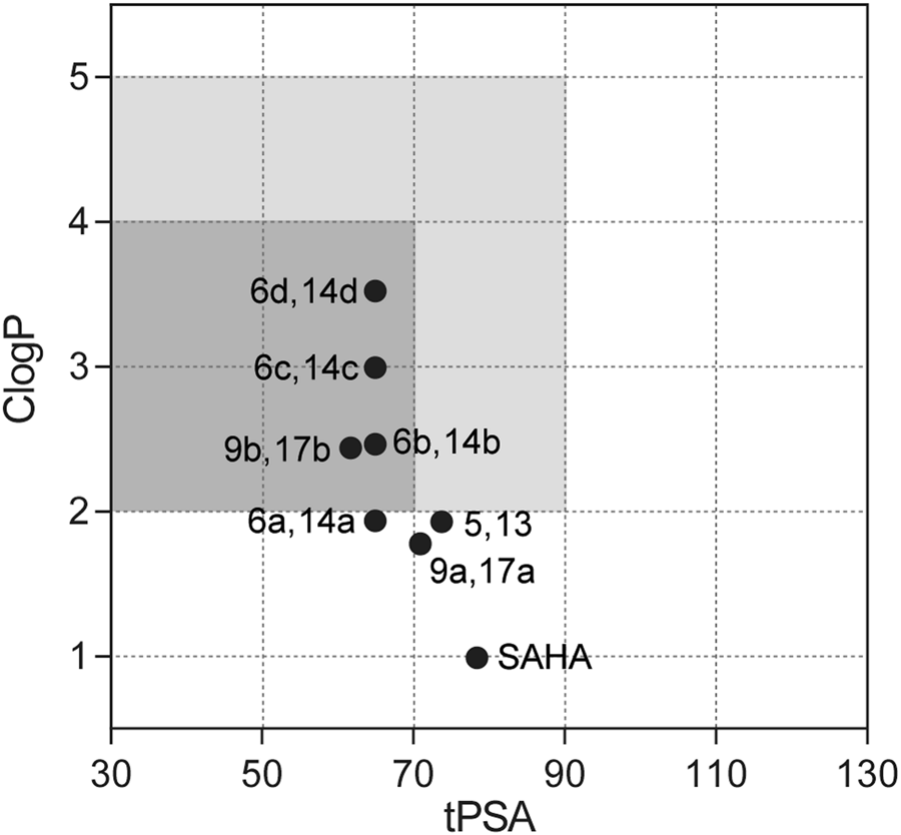
Calculated physiochemical properties of HDAC inhibitors. *Preferred* range for crossing BBB is shown in dark gray color and *suggested* limits for crossing BBB are shown in light gray color. ClogP and tPSA values are calculated by ChemBioDraw Ultra 12.0 software.

Delivering therapeutic agents to the central nervous system (CNS) remains a major challenge for the treatment of brain cancers due to the presence of the blood-brain barrier (BBB). The success of therapeutic agents in treating various brain cancers suggests that it is highly desirable to identify small molecule inhibitors, capable of crossing the BBB. Important molecular property criteria that are widely used to evaluate the ability of compounds to cross the BBB include lipophilicity (clogP), topological polar surface area (tPSA), molecular weight (MW), and number of hydrogen bond donors (HBD)^41^. Therefore, we calculated clogP, tPSA, MW, and clogP of synthesized HDAC inhibitors to evaluate their ability to cross the BBB. MW of all compounds are within the *preferred* limit (MW < 450) and HBD of all compounds except compound **5**, **13** and SAHA are within the *suggested* limit (HBD < 3), while HBD of compound **5**, **13** and SAHA are 3. As shown in Fig. 7, compounds **6b-c**, **9b**, **14b-c**, and **17b** were within the *preferred* range of clogP and tPSA (clogP = 2–4, tPSA < 70 Å^2^), while the reference drug SAHA was not located in the *preferred* or *suggested* range of clogP and tPSA, suggesting that these compounds (**6b-c**, **9b**, **14b-c**, and **17b)** are very likely to cross the BBB and accumulate in the brain, compared to the reference drug SAHA.

### *In vitro* PAMPA-BBB assay

In order to verify BBB permeability of compound **9b** and SAHA, we next carried out the parallel artificial membrane permeability assay of BBB (PAMPA-BBB) (Table 2)^42^. The PAMPA-BBB assay is a powerful *in vitro* technique to give a good prediction on the passive BBB permeability of drugs. A set of structurally diverse drugs were first selected as positive and negative controls, in that progesterone and lidocaine were classified as CNS+ (high brain penetration) and theophylline was classified as CNS- (low brain penetration). The PAMPA-BBB assay demonstrated that the effective permeability value of compound **9b** (*P*_*e*_ = 27.15) was much higher than SAHA (*P*_*e*_ = 2.77), suggesting that compound **9b** could more efficiently penetrate into CNS than SAHA. The reference drugs, progesterone and lidocaine, which are classified as CNS+, showed high effective permeability (*P*_*e*_) values, while theophylline, classified as CNS- furnished a very low effective permeability (*P*_*e*_) value as reported in the literature^43^. These results also illustrated that experimentally determined effective permeability (*P*_*e*_) of compound **9b** and SAHA was in good agreement with the theoretical prediction of BBB permeability shown in Fig. 7.

**Table 2:** Effective permeability (*P*_*e*_) of compound **9b** and SAHA in the PAMPA-BBB assay.

Compounds	*P*_*e*_ (10^−6^ cm/s)^*a*^	CNS +/− classification^*b*^
Progesterone	35.52 ± 0.33	CNS +
Lidocaine	18.98 ± 3.05	CNS +
Theophylline	0.10 ± 0.02	CNS −
**9b**	27.15 ± 1.59	CNS +
SAHA	2.77 ± 0.05	CNS −

^*a*^Data are expressed as the mean ± SD from three independent experiments. ^*b*^CNS + indicates (*P*_*e*_ > 10) and CNS - indicates (*P*_*e*_ < 10).

### *In vivo* brain pharmacokinetic studies

In light of the encouraging *in vitro* PAMPA- BBB data of compound **9b**, we performed *in vivo* brain pharmacokinetic studies. Compound **9b** and SAHA were administered intravenously to male mice at 2 mg/kg and the concentrations of **9b** and SAHA in mice brain and plasma were analyzed using LC-MS/MS at 30 minutes and 1 hour time points. As is apparent from the data shown in Table 3, *in vivo* brain pharmacokinetic studies indicated that compound **9b** displayed a good brain uptake (414.6 ng/mL at 0.5 h and 52.9 ng/mL at 1 h), which is consistent with its calculated permeability properties and *in vitro* PAMPA- BBB data. In contract, FDA-approved HDAC inhibitor, SAHA afforded a poor brain uptake (14.5 ng/mL), which is 29-fold less effective than compound **9b** (414.6 ng/mL) to distribute in brain at 30 minutes after the intravenous administration. Furthermore, the brain to plasma exposure profile for compound **9b** was superior to that obtained for SAHA. Overall, *in vivo* brain pharmacokinetic studies illustrated that compound **9b** effectively distributed into CNS.

**Table 3:** Brain pharmacokinetic studies of compound **9b** and SAHA^*a*^.

Compound	Route	Dose (mg/kg)	Brain concentration (ng/mL)		Plasma concentration (ng/mL)		Brain/plasma ratio	
			0.5 h	1 h	0.5 h	1 h	0.5 h	1 h
**9b**	IV	2	414.6 ± 88.7	52.9 ± 1.6	46.0 ± 13.9	7.3 ± 1.0	9.0	7.2
SAHA	IV	2	14.5 ± 2.8	BQL	39.2 ± 11.5	4.5 ± 2.7	0.4	NA

^*a*^Compound **9b** and SAHA were administrated to ICR male mice by IV route at the dose of 2 mg/kg. Brain samples were collected at 0.5 and 1 hour time points and homogenized at a 1:4 ratio of tissue weight (g) to PBS volume (mL). Plasma samples were collected at 0.5 and 1 hour time points. Aliquots (20 μL) of brain homogenate or plasma samples were mixed with 180 μL of acetonitrile, vortexed, and centrifuged at 15,000 rpm for 5 minutes at 4 °C. The resulting supernatants were used for LC-MS/MS analysis. BQL: below quantifiable limit. NA: not applicable. Data are expressed as the mean ± SD (n = 4).

### Biological evaluation of compound **9b**

**Figure 8:**
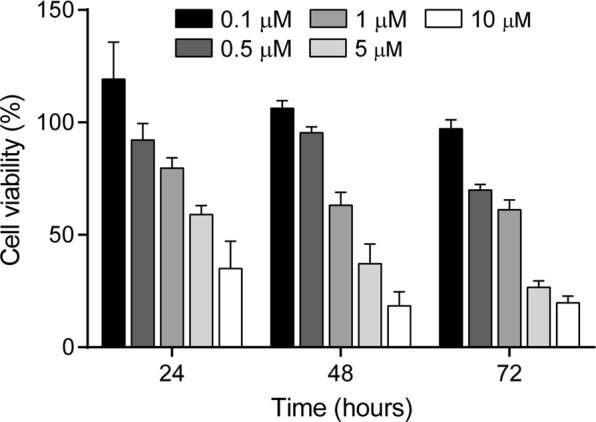
Dose and time-dependent anti-proliferative effect of **9b** against SH-SY5Y cell line. Data are presented as the mean ± SD (n = 4).

We next investigated the dose and time-dependent effect of compound **9b** on the growth of SH-SY5Y cells. SH-SY5Y cells were treated with the indicated concentrations of compound **9b** for 24, 48, and 72 hours and cell viability was measured by MTS colorimetric assay (Fig. 8). The data indicated that compound **9b** exhibited a potent anti-proliferative activity against SH-SY5Y in a dose and time-dependent manner. Compound **9b** impaired nearly 73% of SH-SY5Y cell growth at the concentration of 5 μM for 72 hours.

**Figure 9:**
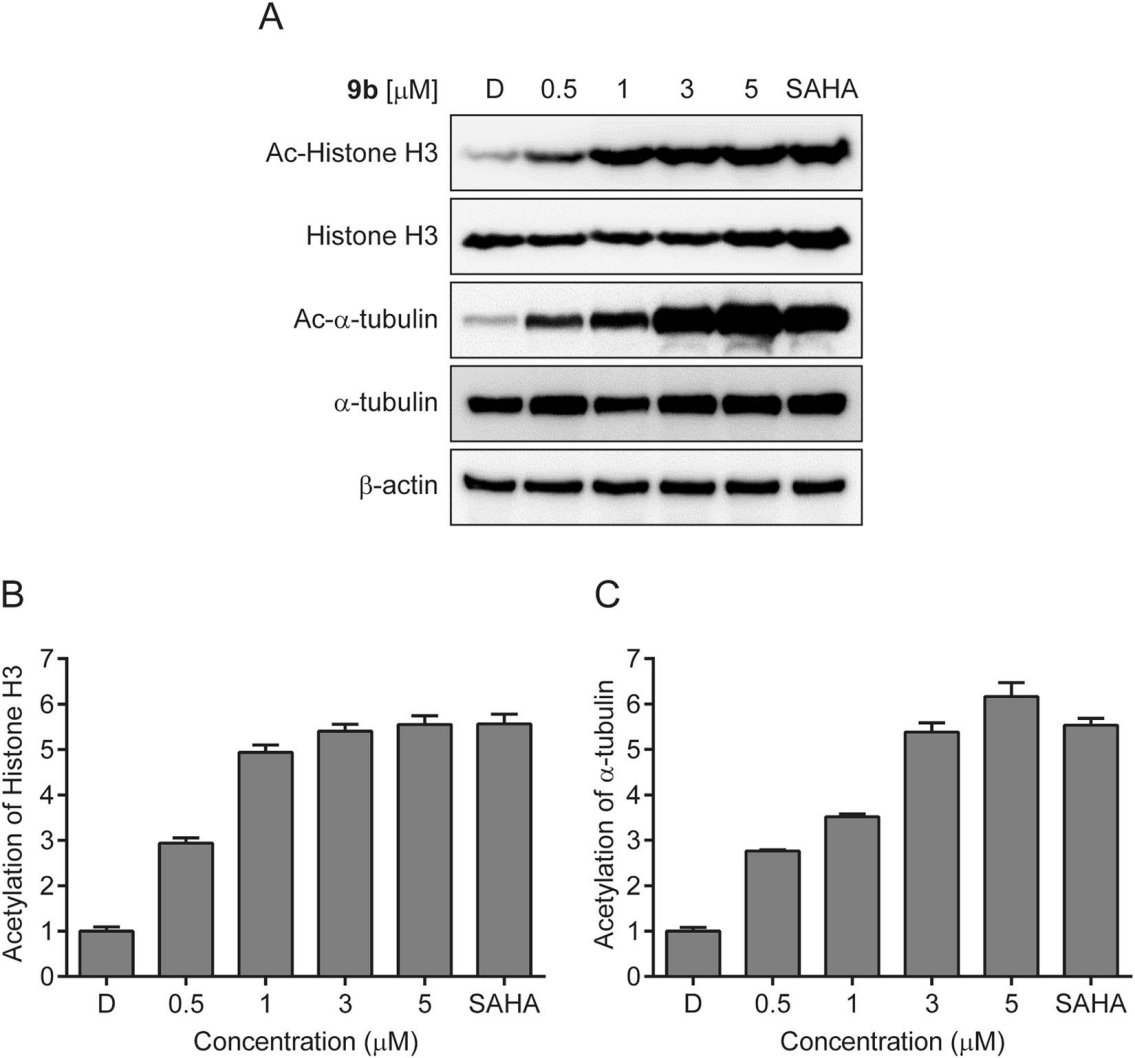
(**A**) Effect of compound **9b** on the acetylation status of Histone H3 and *α*-tubulin. SH-SY5Y cells were incubated with the indicated concentrations of **9b** for 24 h and the acetylation status of Histone H3 and *α*-tubulin was measured by western blot. DMSO (**D**) and SAHA (1 μM) were employed as a negative and a positive control, respectively. (**B**) Densitometry analysis of Ac-Histone H3 and (**C**) Ac-*α*-tubulin. Fold increase of Ac-Histone H3 and Ac-*α*-tubulin were analyzed using Image J software. Control value = 1. Data are presented as the mean ± SD (n = 3).

To investigate whether compound **9b** exhibited its anticancer effect through HDACs inhibition, we next studied the cellular biomarkers of HDACs inhibition (Fig. 9). Histone H3 and *α*-tubulin are well-documented substrates of HDAC1 and HDAC6, respectively. Thus, the acetyl groups of those substrates can be epigenetically removed by HDAC1 and HDAC6. As shown in Fig. 9, treatment of SH-SY5Y cells with compound **9b** significantly induced the accumulation of acetylated Histone H3 and *α*-tubulin in a dose-dependent manner, indicating that compound **9b** inhibited the deacetylase activities of HDAC1 and HDAC6. Treatment of SH-SY5Y cells even with 0.5 μM concentration of **9b** promoted the acetylation of Histone H3 and *α*-tubulin *via* inhibiting HDAC1 and HDAC6, respectively. Interestingly, 1 μM concentration of **9b** caused the dramatic increase of acetylated Histone H3, suggesting that the deacetylase activity of HDAC1 was significantly inhibited even with the administration of **9b** at 1 μM concentration. In contrast, the acetylation of *α*-tubulin dose-dependently increased in proportion to the concentration of compound **9b** up to 5 μM concentration, indicating that inhibition of HDAC6 enzyme was not fully completed at least up to 3 μM concentration in this cell-based assay. Nonetheless, the results suggested that compound **9b** suppressed HDAC1 and HDAC6 activities in a dose-dependent manner and exerted its anticancer effect via those HDACs inhibition.

We next examined the inhibitory activity of compound **9b** against HDAC1, 3, 6, and 7 isoforms. For comparison, the clinically approved HDAC inhibitor SAHA was tested as a reference drug. As shown in Table 4, compound **9b** very potently inhibited HDAC class I (HDAC1 IC_50_ = 84.9 nM, HDAC3 IC_50_ = 142.2 nM) and class IIa (HDAC6 IC_50_ = 95.9 nM) enzymes, but did not inhibited class IIb (HDAC7 IC_50_ = 3,036 nM) enzyme, efficiently. Similarly, the reference drug SAHA inhibited HDAC1, HDAC3, HDAC6, and HDAC7 enzymes with IC_50_ values of 102.7 nM, 61.1 nM, 198.5 nM, and 29,290 nM, respectively. Importantly, compound **9b** maintained low nanomolar IC_50_ values in the 85–96 nM range with HDAC1 and HDAC6, better than SAHA (103–199 nM). The IC_50_ values of compound **9b** and SAHA against HDAC6 are not completely correlated with the results in Western blotting experiment, shown in Fig. 9, in that SAHA (1 μM) induced more acetylation of *α*-tubulin than compound **9b** (1 μM). Nonetheless, it is worth noting that compound **9b** displayed good inhibitory activities toward class I and IIa HDACs, which are considered to play an important role in cancer cell proliferation, survival, and metastasis. Furthermore, these HDAC inhibition activity of compound **9b** and SAHA well correlated with their anti-proliferative activity, in that compound **9b** (GI_50_ = 2.01 μM) was measured more potent than SAHA (GI_50_ = 2.90 μM) (Table 1).

**Table 4:** HDAC inhibition activity of compound **9b** and SAHA^*a*^.

Class	Enzymes	9b (IC_50_; nM)	SAHA (IC_50_; nM)
I	HDAC1	84.9 ± 25.1	102.7 ± 5.9
	HDAC3	142.2 ± 45.7	61.1 ± 1.54
IIa	HDAC7	3,036 ± 788.5	29,290 ± 1,325
IIb	HDAC6	95.9 ± 0.78	198.5 ± 103.0

^*a*^Data are presented as the mean ± SD (n = 2).

**Figure 10:**
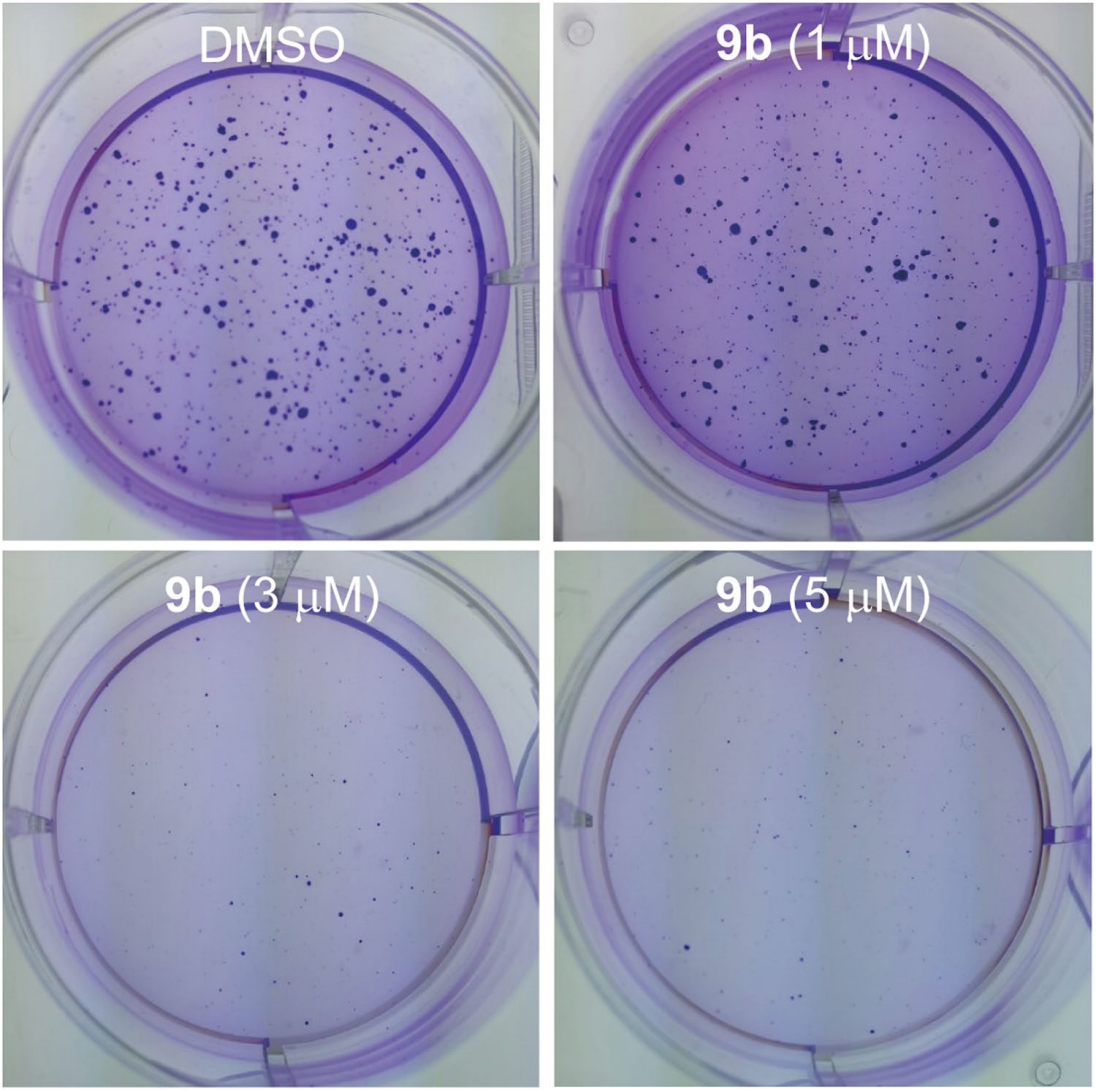
Suppressive effect of compound **9b** on colony formation of SH-SY5Y neuroblastoma. The cells were seeded into 6-well plates and cultured for 3 weeks, followed by Crystal Violet staining.

Anchorage-independent cell growth is a hallmark of cell carcinogenesis and indicates the ability of transformed cells to grow independently of a solid surface. The soft agar colony formation assay is a widely used method for measuring the capability of anchorage-independent cell growth *in vitro*^44^. Hence, we examined the suppressive effect of compound **9b** on anchorage-independent cell growth by the soft agar colony formation assay (Fig. 10). Treatment of SH-SY5Y cells with **9b** exhibited inhibitory effect on colony formation of SH-SY5Y neuroblastoma. Moreover, the colony formation capacity of SH-SY5Y cells decreased dose-dependently with the increase of **9b** concentration. 3 μM concentration of compound **9b** almost completely inhibited the anchorage-independent cell growth of SH-SY5Y neuroblastoma. Overall, the data suggested that compound **9b** effectively suppressed the ability of SH-SY5Y cells to grow anchorage-independently in a dose-dependent manner.

### Molecular modeling of compound **9b**

The crystal structures of HDAC enzymes are characterized by the active Zn^2+^ ion in the bottom of the pocket, the hydrophobic channel reaching to the active Zn^2+^ ion, and the surface rim at the entrance of the pocket.

**Figure 11:**
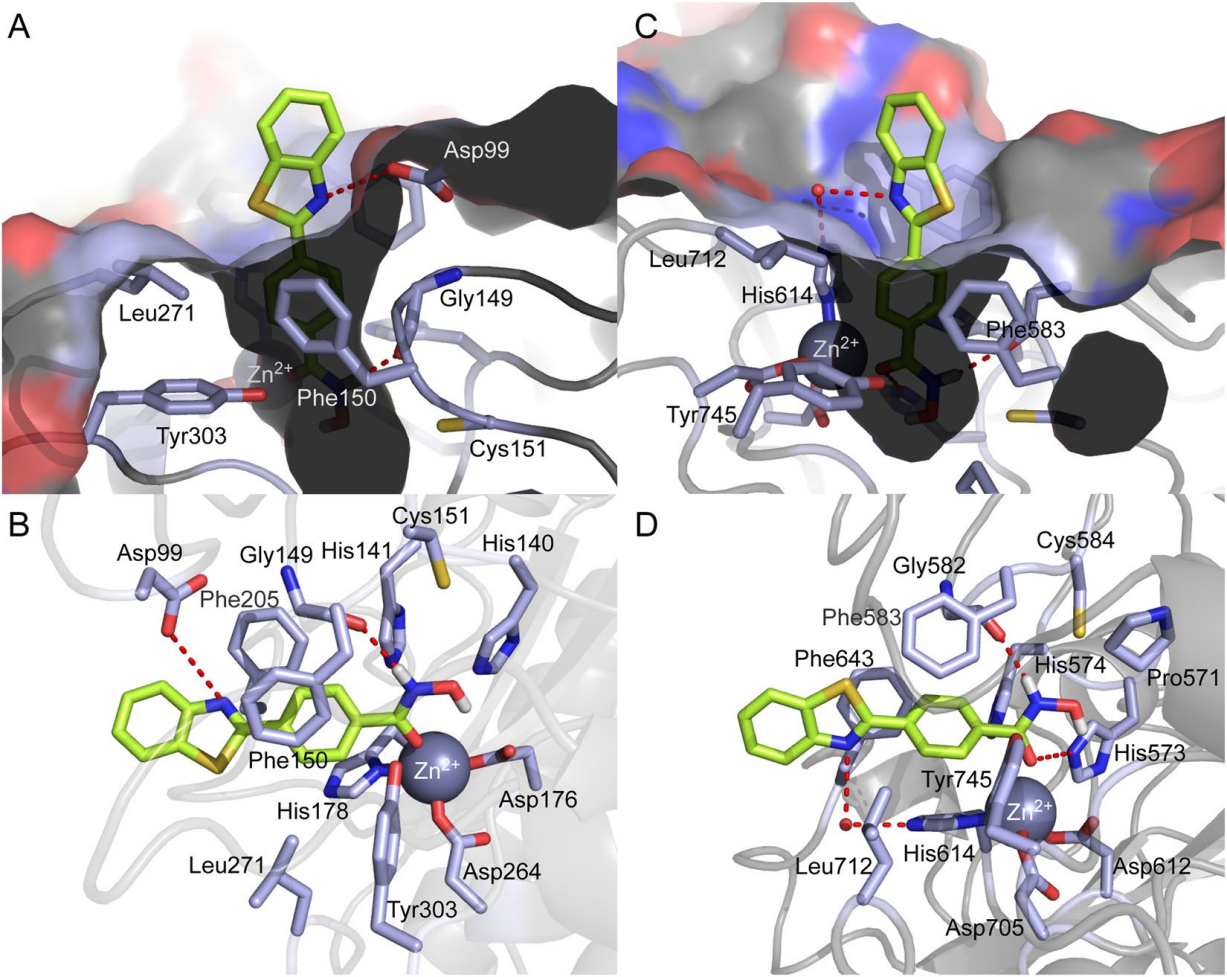
Docking pose of **9b** in the binding site of HDAC1 (PDB code: 4BKX) and HDAC6 (PDB code: 5EF7). (**A**) Surface representation of HDAC1 and **9b** complex. (**B**) Cartoon and sticks representation of HDAC1 and **9b** complex. (**C**) Surface representation of HDAC6 and **9b** complex. (**D**) Cartoon and sticks representation of HDAC6 and **9b** complex. The carbon, oxygen, nitrogen, and sulfur atoms of **9b** are shown in lime, red, blue, and yellow, respectively. The side chains of the binding site are colored according to the atom type (carbon, light blue; oxygen, red; nitrogen, blue) and are labeled with their residue name. The hydrogen bond is shown as a dashed line. Molecular docking simulations were performed by Autodock 4.2 and docking poses were visualized using PyMOL1.3.

To assess the precise binding pose of **9b** in the binding pocket of HDAC1 (PDB code: 4BKX) and HDAC6 (PDB code: 5EF7), we performed a molecular docking study (Fig. 11). *In silico* docking study displayed that the hydroxamic acid group of **9b** chelated the Zn^2+^ ion in the bottom of the pocket in a monodentate fashion using its carbonyl oxygen atoms in both HDAC1 and HDAC6 isoforms. Although a majority of HDAC inhibitors conferred a canonical bidentate Zn^2+^ coordination geometry, several ligands in complex with HDACs have recently been reported to display the similar monodentate Zn^2+^ coordination, illustrating that the capping and linker units attached to the hydroxamate affect its degree of coordination to Zn^2+^, resulting in either monodentate or bidentate chelation^27,45^. The hydroxamate NH group of **9b** formed an additional hydrogen bonding interaction with Gly149 residue of HDAC1 and Gly582 residue of HDAC6 in a similar fashion. Moreover, the middle phenyl ring of **9b** fitted into the hydrophobic channel, forming π-π stacking interactions with the lipophilic Phe150 and Phe205 resides of HDAC1, and Phe583 and Phe643 residues of HDAC6, occupying the hydrophobic channels of HDAC1 and HDAC6, respectively. However, we observed that the orientation of the benzothiazole capping group in HDAC1 was different from HDAC6 isoform. The docking pose of **9b** to HDAC1 indicated that the nitrogen atom of the benzothiazole formed a potential hydrogen bonding interaction with the side chain of Asp99 at the rim of the pocket and sulfur atom formed proximal Van der Waals interactions with Leu271 and Phe150. On the contrary, the docking of **9b** in HDAC6 suggested that the nitrogen atom of the benzothiazole formed a hydrogen bonding interaction with a conserved water molecule, which interacted with zinc ligand H614. Nonetheless, this docking study acknowledged the inhibitory potential of **9b** against both HDAC1 and HDAC6 isoforms.

## Conclusion

In summary, we designed and synthesized a new series of CNS penetrant HDAC inhibitors. Biological evaluation of these HDAC inhibitors indicated that benzothiazole analogue **9b** exerted the most potent anti-proliferative activity (IC_50_ = 2.01 μM) against human neuroblastoma SH-SY5Y cell line, slightly better than the clinically approved HDAC inhibitor, SAHA (IC_50_ = 2.90 μM). The exposure of SH-SY5Y cells with compound **9b** significantly induced the accumulation of acetylated Histone H3 and *α*-tubulin, which are characteristic cellular biomarkers of HDACs inhibition. HDACs enzyme assay further disclosed that compound **9b** efficiently inhibited HDAC1 and HDAC6 isoforms with IC_50_ values of 84.9 nM and 95.9 nM, respectively. The soft agar assay, which is an *in vitro* method for measuring the capability of anchorage-independent cell growth, clearly suggested that compound **9b** suppressed colony formation of SH-SY5Y cells. The theoretical prediction, *in vitro* PAMPA-BBB assay, and *in vivo* brain pharmacokinetic studies confirmed that compound **9b** had much higher brain uptake than SAHA. The docking study revealed that compound **9b** occupied the binding pocket of HDAC1 and HDAC6 enzymes with monodentate zinc ion chelation, hydrogen bonding and lipophilic π-π interactions. Collectively, compound **9b** represents a new class of CNS penetrant HDAC inhibitor and demonstrates therapeutic potential for the treatment of brain cancers and CNS disorders.

## Methods

### Synthesis

The synthesis and physicochemical properties of compounds are provided as supplementary information available with this article online.

### Docking studies

*In silico* docking of compound **9b** with the 3D coordinates of the X-ray crystal structures of HDAC1 (PDB code: 4BKX) or HDAC6 (PDB code: 5EF7) was accomplished using the AutoDock 4.2 program downloaded from the Molecular Graphics Laboratory of the Scripps Research Institute. The AutoDock program was chosen because it uses a genetic algorithm to generate the poses of the ligand inside a known or predicted binding site utilizing the Lamarckian version of the genetic algorithm where the changes in conformations adopted by molecules after *in situ* optimization are used as subsequent poses for the offspring. In the docking experiments carried out, Gasteiger charges were placed on the X-ray structures of HDACs along with **9b** using tools from the AutoDock suite. A grid box centered on the substrate binding pocket of HDACs enzyme with definitions of 60 × 60 × 60 points and 0.375 Å spacing was chosen for ligand docking experiments. The docking parameters consisted of setting the population size to 150, the number of generations to 27,000, and the number of evaluations to 2,500,000, while the number of docking runs was set to 100 with a cutoff of 1 Å for the root-mean-square tolerance for the grouping of each docking run. The docking pose of HDAC1 or HDAC6 with compound **9b** was depicted in Fig. 11 and rendering of the picture was generated using PyMOL1.3 (DeLanoScientific).

### Cell culture

SH-SY5Y cells were grown in DMEM with L-glutamin supplemented with streptomycin (500 mg/mL), penicillin (100 units/mL), and 10% fetal bovine serum (FBS). Cells were grown to confluence in a humidified atmosphere (37 °C, 5% CO_2_).

### Cell proliferation assay

SH-SY5Y cells (1.5 × 10^3^ cells/well) were seeded in a clear 96-well plate, the medium volume was brought to 100 µL, and the cells were allowed to attach overnight. The next day, various concentrations of compounds or DMSO were added to the wells. Cells were then incubated at 37 °C for 24, 48 and 72 h. Cell viability was determined using the Promega Cell Titer 96 Aqueous One Solution cell proliferation assay. Absorbance at 490 nm was read on Tecan Infinite F200 Pro plate reader, and values were expressed as percent of absorbance from cells incubated in DMSO alone.

### Western blot

Cells were seeded in 100 mm culture dishes (1 × 10^6^ cells/dish), and allowed to attach overnight. Inhibitors were added at the concentrations as indicated and the cells were incubated for an additional 24 hours. For comparison, cells were also incubated with DMSO (0.5%) for 24 hours. Cells were harvested in ice-cold lysis buffer (23 mM Tris-HCl pH 7.6, 130 mM NaCl, 1% NP-40, 1% sodium deoxycholate, 0.1% SDS), and 30 µg of lysate per lane was separated by SDS-PAGE and followed by transferring to a PVDF membrane (Bio-Rad). The membrane was blocked with 5% skim milk in TBST, and then incubated with the corresponding antibody (Ac-α-tubulin, α-tubulin, Ac-Histone H3, Histone H3 or β-actin). After binding of an appropriate secondary antibody coupled to horseradish peroxidase, proteins were visualized by ECL chemiluminescence according to the instructions of the manufacturer (GE healthcare, USA).

### HDAC activity assay

HDAC1, 3, 6 and 7 activity assays were performed according to the manufacturer’s protocol (BPS Biosciences). Briefly, enzymes were incubated with various concentrations of compound **9b** and SAHA at 37 °C for 30 min in the presence of an HDAC fluorimetric substrate. The HDAC assay developer was added to the mixture and incubated the plate at rt for 15 min. The fluorescence intensity was measured using a Tecan Infinite F200 Pro plate reader.

### Colony formation assay

Cells (10^4^ cells/well) were seeded in 6 well plate with 1.2% agar. After 12 h incubation, cells were treated with the indicated concentrations of compound **9b** or DMSO at 37 °C in 5% humidified CO_2_ for 3 weeks with continuously changing medium every week. After medium was thrown away, colonies were stained with 0.05% crystal violet for 10 min.

### BBB-PAMPA procedures

BBB-PAMPA was conducted by manufacturer’s instruction (pION Inc, MA, USA). Briefly, test compound was diluted in donor buffer (pH 7.4) to be 25 μM and add 200 μL in lower bottom of 96 well PAMPA sandwich plate. Transmembrane side to donor part was coated with BBB lipid solution and add 200 μL acceptor buffer in upper part of PAMPA sandwich plate. After incubation for 4 h at 25 °C, each part of samples was transferred to new U.V plate then measured U.V spectra at wavelength from 250 nm to 498 nm and permeability rate (*P*_*e*_, 10^−6^ cm/sec) was analyzed using pION PAMPA Explorer software (ver3.8).

### Brain pharmacokinetic studies

The study was approved by the Institutional Animal Care and Use of Committee of Daegu-Gyeongbuk Medical Innovation Foundation (DGMIF) and performed in accordance with protocols approved by the Institutional Animal Care and Use of Committee. Compound **9b** and SAHA were intravenously administered to ICR Male mice, aged 7–8 weeks and weighing 25–30 g at 2 mg/kg with solution formulation of 10% DMSO, 70% PEG400, and 20% saline. Brain and plasma samples were collected at 0.5 and 1 h time points. Brain samples were homogenized at a 1:4 ratio of sample weight (g) to PBS volume (mL). Aliquots (20 μL) of brain homogenate were mixed with 180 μL of acetonitrile, vortexed and centrifuged at 15,000 rpm for 5 minutes at 4 °C. The resulting supernatants were used for LC-MS/MS analysis. Mice plasma samples were collected at 0.5 and 1 h time points. 180 μL of acetonitrile was added to 20 μL of plasma samples, vortexed and centrifuged at 15,000 rpm for 5 minutes at 4 °C. Concentrations of compound **9b** and SAHA in mice brain and plasma were determined using an LC-MS/MS. The data found (ng/mL) was multiplied by its dilution factors to obtain the concentration (ng/mL) of compound **9b** and SAHA in brain and plasma.

## Supplementary information

Supplementary information
