# Supplementary material for: Design, synthesis and biological evaluation of a series of CNS penetrant HDAC inhibitors structurally derived from amyloid-β probes
Source: Sci Rep. 2019 Sep 12;9:13187. doi: 10.1038/s41598-019-49784-9 (PMC6742641; doi:10.1038/s41598-019-49784-9)
Supplement: Supplementary file 1 — Supplementary information [file 41598_2019_49784_MOESM1_ESM.docx]

**Supplementary Information**

Design, synthesis and biological evaluation of a series of CNS penetrant HDAC inhibitors structurally derived from amyloid-*β* probes

Myeong A Choi, Sun You Park, Hye Yun Chae, Yoojin Song, Chiranjeev Sharma,

Young Ho Seo*

*College of Pharmacy, Keimyung University, Daegu 704-701, South Korea.*

* To whom correspondence should be addressed. E-mail: [seoyho@kmu.ac.kr](mailto:seoyho@kmu.ac.kr)


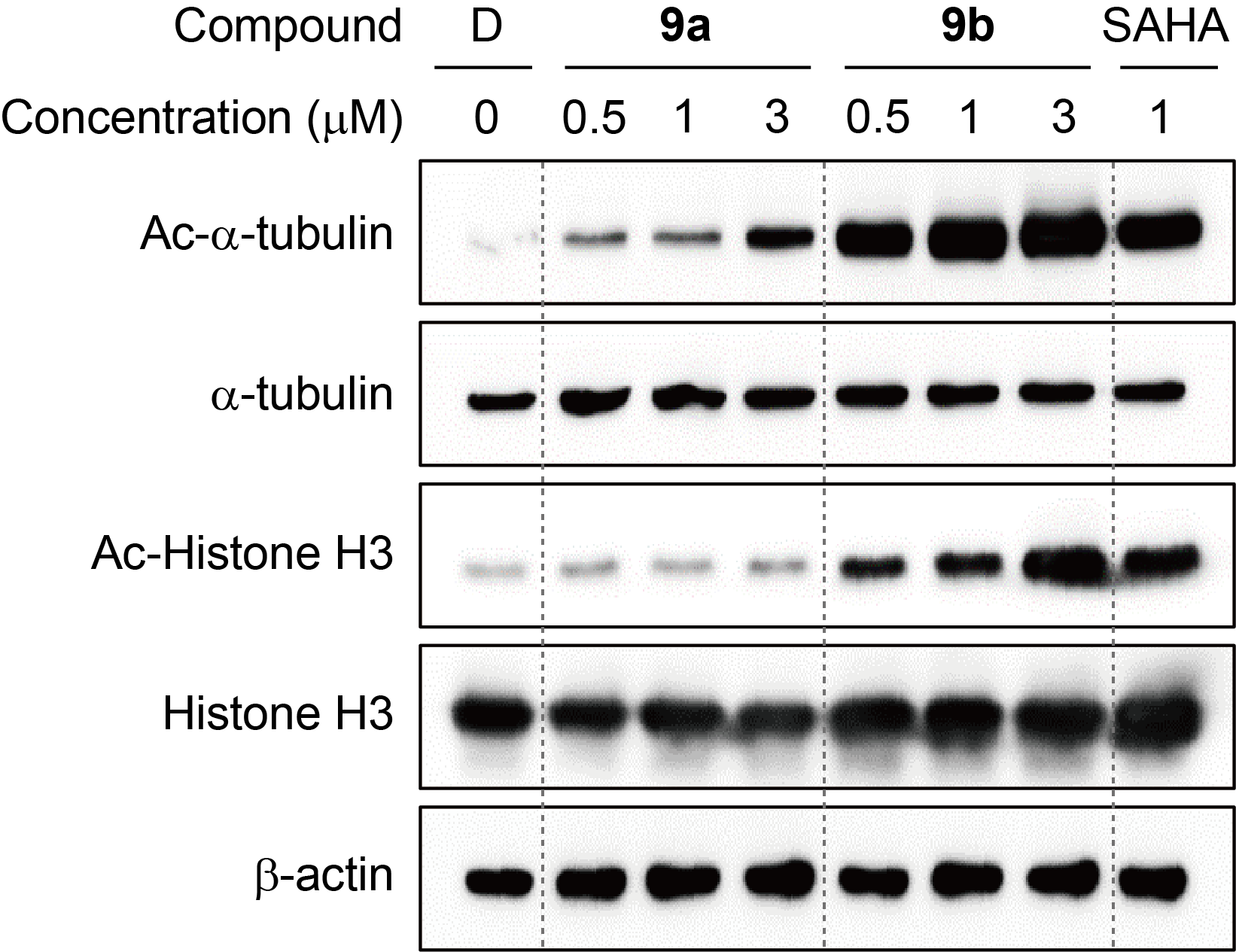


**Figure S1**. Comparative effect of compound **9a** and **9b** on the acetylation status of *α*-tubulin and Histone H3. SH-SY5Y cells were incubated with the indicated concentrations of compound **9a** and **9b** for 24 h and the acetylation status of *α*-tubulin and Histone H3 was measured by western blot. DMSO (D) and SAHA (1 μM) were employed as a negative and a positive control, respectively.

**1. Synthesis**

*1.1. Chemistry*

*1.1.1. General methods and materials*

All reagents and solvents were purchased from commercial suppliers and used without further purification. All experiments dealing with moisture-sensitive compounds were carried out under argon atmosphere. Concentration or solvent removal under reduced pressure was carried out using rotary evaporator. Analytical thin layer chroatography was performed on precoated silica gel F_254_ TLC plates (E, Merck) with visualization under UV light or by staining using iodine. Column chromatography was conducted under medium pressure on silica (Merck Silica Gel 40-63 m) or performed by medium pressure liquid chromatography (MPLC, Biotage Isolera One instrument) with prepacked silica gel cartridges (Biotage SNAP HP-Sil). NMR analyses were carried out using a JNM-ECZ500R (500 MHz) manufactured by Jeol resonance. Chemical shifts are reported in parts per million (*δ*). The deuterium lock signal of the sample solvent was used as a reference, and coupling constants (*J*) are given in hertz (Hz). The splitting pattern abbreviations are as follows: s, singlet; d, doublet; t, triplet; q, quartet; dd, doublet of doublet; td, triplet of doublet; m, multiplet. The purities of all final compounds were confirmed to be higher than 95% by analytical HPLC performed with a dual pump Shimadzu LC-6AD system equipped with VP-ODS C18 column (4.6 mm × 250 mm, 5 μm, Shimadzu). MS analysis was performed using an Agilent 6530 Accurate-Mass Q-TOF LC/MS system with Agilent 1290 Infinity LC (Agilent technologies, Santa Clara, CA, USA).

*1.1.2. Methyl 4-(1H-benzo[d]imidazol-2-yl)benzoate (****3****)*

A mixture of *o*-phenylenediamine (5.84 g, 53.98 mmol) and methyl 4-formylbenzoate (9.75 mL, 59.37 mmol) in DMF (180 mL) and water (20 mL) was stirred at 80 ^o^C for 36 h in an open flask. After completion of reaction, the mixture was concentrated under reduced pressure and purified by MPLC to afford compound **3** in 88% yield. R_f_ = 0.23 (3:7 ethyl acetate: hexanes). ^1^H NMR (500 MHz, CD_3_OD) δ 8.19-8.14 (m, 4H), 7.63 (s, 2H), 7.30-7.27 (m, 2H), 3.93 (s, 3H). ^13^C NMR (125 MHz, CD_3_OD) δ 166.5, 150.6, 133.9, 131.2, 129.9, 126.4, 123.1, 51.5.

*1.1.3. General procedure for the synthesis of compounds (****4a-d*** *and* ***12a-d****)*

Compound **3** or **11** (1.25 mmol) in DMF (5 mL) was added to sodium hydride (2.25 mmol) in DMF (5 mL) at 0 °C under argon atmosphere. After the mixture was stirred at rt for 2 h, the corresponding alkyl iodide (1.38 mmol) was added to the reaction mixture at rt. After being stirred for another 8 h, the reaction mixture was diluted with ethyl acetate, washed with water, dried over Na_2_SO_4_, concentrated under reduced pressure, and purified by MPLC to afford compound **4a-d** or **12a-d** in 11-91%.

*1.1.3.1. Methyl 4-(1-methyl-1H-benzo[d]imidazol-2-yl)benzoate (****4a****).* 70% yield. R_f_ = 0.26 (3:7 ethyl acetate: hexanes). ^1^H NMR (500 MHz, CDCl_3_) δ 8.17 (d, *J* = 8.5 Hz, 2H), 7.85-7.81 (m, 3H), 7.38-7.36 (m, 1H), 7.33-7.30 (m, 2H), 3.94 (s, 3H), 3.84 (s, 3H). ^13^C NMR (125 MHz, CDCl_3_) δ 166.5, 152.5, 143.0, 136.7, 134.5, 131.1, 129.9, 129.4, 123.3, 122.7, 120.1, 109.8, 52.4, 31.8.

*1.1.3.2. Methyl 4-(1-ethyl-1H-benzo[d]imidazol-2-yl)benzoate (****4b****).* 49 % yield. R_f_ = 0.25 (3:7 ethyl acetate: hexanes). ^1^H NMR (500 MHz, CDCl_3_) δ 8.19 (d, *J* = 8.5 Hz, 2H), 7.85-7.81 (m, 3H), 7.45-7.43 (m, 1H), 7.35-7.31 (m, 2H), 4.32-4.27 (m, 2H), 3.96 (s, 3H), 1.47 (t, *J* = 7.0 Hz, 3H). ^13^C NMR (125 MHz, CDCl_3_) δ 166.6, 152.3, 143.3, 135.6, 134.9, 131.2, 130.0, 129.3, 123.2, 122.7, 120.3, 110.1, 52.4, 39.8, 15.4. ESI MS (*m/z*) 281.12 [M+H]^+^.

*1.1.3.3. Methyl 4-(1-propyl-1H-benzo[d]imidazol-2-yl)benzoate (****4c****).* 45% yield. R_f_ = 0.30 (3:7 ethyl acetate: hexanes). ^1^H NMR (500 MHz, CDCl_3_) δ 8.19 (d, *J* = 8.5 Hz, 2H), 7.83-7.79 (m, 3H), 7.42-7.40 (m, 1H), 7.33-7.29 (m, 2H), 4.19 (t, *J* = 7.5 Hz, 2H), 3.95 (s, 3H), 1.85-1.80 (m, 2H), 0.84 (t, *J* = 7.1 Hz, 3H). ^13^C NMR (125 MHz, CDCl_3_) δ 166.5, 152.5, 143.0, 136.7, 134.5, 131.1, 129.9, 129.4, 123.3, 122.7, 120.1, 109.8, 52.4, 31.8, 29.7, 15.4. ESI MS (*m/z*) 295.14 [M+H]^+^.

*1.1.3.4. Methyl 4-(1-butyl-1H-benzo[d]imidazol-2-yl)benzoate (****4d****).* 46 % yield. R_f_ = 0.35 (3:7 ethyl acetate: hexanes). ^1^H NMR (500 MHz, CDCl_3_) δ 8.17 (d, *J* = 8.5 Hz, 2H), 7.85-7.81 (m, 3H), 7.38-7.36 (m, 1H), 7.33-7.30 (m, 2H), 4.20 (t, *J* = 7.5 Hz, 2H), 3.94 (s, 3H), 1.81-1.74 (m, 2H), 1.24-1.22 (m, 2H), 0.83 (t, *J* = 7.5 Hz, 3H). ^13^C NMR (125 MHz, CDCl_3_) δ 166.5, 152.5, 143.2, 135.8, 135.1, 131.1, 129.9, 129.4, 123.1, 122.7, 120.1, 110.3, 52.4, 44.6, 31.9, 19.9, 13.5.

*1.1.3.5. Methyl 3-(1-methyl-1H-benzo[d]imidazol-2-yl)benzoate (****12a****).* 91% yield. R_f_ = 0.25 (4:6 ethyl acetate: hexanes). ^1^H NMR (500 MHz, CDCl_3_) δ 8.44 (s, 1H), 8.19 (d, *J* = 8.0 Hz, 1H), 8.02 (d, *J* = 7.5 Hz, 1H), 7.85-7.83 (m, 1H), 7.63 (t, *J* = 8.0 Hz, 1H), 7,43-7.41 (m, 1H), 7.37-7.33 (m, 2H), 4.0 (s, 3H), 3.90 (s, 3H). ^13^C NMR (125 MHz, CDCl_3_) δ 166.6, 152.8, 143.0, 134.0, 130.9, 130.8, 130.4, 129.2, 123.2, 122.8, 120.1, 109.9, 52.5, 31.96. ESI MS (*m/z*) 267.11 [M+H]^+^.

*1.1.3.6. Methyl 3-(1-ethyl-1H-benzo[d]imidazol-2-yl)benzoate (****12b****).* 11% yield. R_f_ = 0.28 (4: 6 ethyl acetate: hexanes). ^1^H NMR (500 MHz, CDCl_3_) δ 8.41 (s, 1H), 8.19 (d, *J* = 8.0 Hz, 1H), 7.97 (d, *J* = 8.0 Hz, 1H), 7.84 (d, *J* = 8.5 Hz, 1H), 7.63 (t, *J* = 8.0 Hz, 1H), 7.45 (d, *J* = 9.5 Hz, 1H), 7.36-7.26 (m, 2H), 4.31 (q, *J* = 7.5 Hz, 2H), 3.96 (s, 3H), 1.50 (t, *J* = 7.0 Hz, 3H). ^13^C NMR (125 MHz, CDCl_3_) δ 166.6, 152.4, 143.3, 135.5, 133.9, 131.1, 131.9, 130.8, 130.3, 129.2, 123.1, 122.7, 120.3, 110.2, 52.5, 39.9, 15.4. ESI MS (*m/z*) 281.13 [M+H]^+^.

*1.1.3.7. Methyl 3-(1-propyl-1H-benzo[d]imidazol-2-yl)benzoate (****12c****).* 28 % yield. R_f_ = 0.32 (3:7 ethyl acetate: hexanes). ^1^H NMR (500 MHz, CDCl_3_) δ 8.38 (t, *J* = 1.0 Hz, 1H), 8.17 (td, *J* = 8.0 Hz, 1.0 Hz, 1H), 7.94 (td, *J* = 7.5 Hz, 2.0 Hz, 1H), 7.83-7.81 (m, 1H), 7.59 (t, *J* = 8.0 Hz, 1H), 7.42-7.40 (m, 1H), 7.31-7.29 (m, 2H), 4.19 (t, *J* = 7.5 Hz, 2H), 3.93 (s, 3H), 1.87-1.82 (m, 2H), 0.85 (t, *J* = 7.0 Hz, 3H). ^13^C NMR (125 MHz, CDCl_3_) δ 166.4, 152.6, 143.1, 135.7, 133.9, 131.1, 130.7, 130.2, 129.1, 123.0, 122.5, 120.1, 110.3, 52.4, 46.4, 23.3, 11.2. ESI MS (*m/z*) 295.14 [M+H]^+^.

*1.1.3.8. Methyl 3-(1-butyl-1H-benzo[d]imidazol-2-yl)benzoate (****12d****).* 79 % yield. R_f_ = 0.33 (3:7 ethyl acetate: hexanes). ^1^H NMR (500 MHz, CDCl_3_) δ 8.39 (t, *J* = 1.5 Hz, 1H), 8.17 (td, *J* = 7.5 Hz, 1.5 Hz, 1H), 7.95 (td, *J* = 8.0 Hz, 1.5 Hz, 1H), 7.83-7.81 (m, 1H), 7.60 (t, *J* = 8.0 Hz, 1H), 7.43-7.41 (m, 1H), 7.32-7.28 (m, 2H), 4.23 (t, *J* = 7.5 Hz, 2H), 3.93 (s, 3H), 1.84-1.78 (m, 2H), 1.31-1.24 (m, 2H), 0.85 (t, *J* = 7.5 Hz, 3H). ^13^C NMR (125 MHz, CDCl_3_) δ 166.4, 152.5, 143.1, 135.7, 133.9, 131.1, 130.8, 130.7, 130.2, 129.1, 123.0, 122.7, 120.1, 110.3, 52.4, 44.7, 31.9, 20.0, 13.6. ESI MS (*m/z*) 309.16 [M+H]^+^.

*1.1.4. General procedure for the synthesis of compounds (****5****,* ***6a-d****,* ***9a-b****,* ***13****,* ***14a-d****, and* ***17a-b****)*

A hydroxylamine solution was prepared as follows. Hydroxylamine hydrochloride (3.72 g, 53.5 mmol) in methanol (5 mL) was added to a solution of potassium hydroxide (3.00 g, 53.5 mmol) in methanol (5 mL) at 0 ^o^C and the mixture was stirred for 15 min at 0 ^o^C. After the precipitated potassium chloride was filtered off, the filtrate solution was used for the reaction. A freshly prepared hydroxylamine solution (10 mL) was added to compound **3**, **4a-d**, **8a-b**, **11**, **12a-d**, or **16a-b** (1.2 mmol) in THF (10 mL) at 0 ^o^C. After being stirred for 2 h, the reaction mixture was neutralized with 3*N* HCl to pH 7, diluted with water, and extracted with ethyl acetate. The organic layer was dried over Na_2_SO_4,_ concentrated under reduced pressure, and purified by MPLC to afford compound **5**, **6a-d**, **9a-b**, **13**, **14a-d**, or **17a-b** in 33-55% yield.

*1.1.4.1. 4-(1H-benzo[d]imidazol-2-yl)-N-hydroxybenzamide (****5****).* 40% yield. R_f_ = 0.08 (9:1 ethyl acetate: hexanes). ^1^H NMR (500 MHz, DMSO-D_6_) δ 8.13 (d, *J* = 8.5 Hz, 2H), 7.89 (d, *J* = 8.0 Hz, 2H), 7.59-7.57 (m, 2H), 7.20-7.18 (m, 2H). ^13^C NMR (125 MHz, DMSO-D_6_) δ 163.3, 151.2, 137.2, 130.4, 126.6, 125.9, 122.1. ESI MS (*m/z*) 254.09 [M+H]^+^.

*1.1.4.2. N-hydroxy-4-(1-methyl-1H-benzo[d]imidazol-2-yl)benzamide (****6a****).* 36% yield. R_f_ = 0.17 (9:1 ethyl acetate: hexanes). ^1^H NMR (500 MHz, DMSO-D6) δ 11.41 (s, 1H), 9.19 (s, 1H), 7.95 (dd, J = 10.3, 8.6 Hz, 4H), 7.67 (dd, J = 29.8, 8.0 Hz, 2H), 7.29 (dt, J = 27.1, 7.4 Hz, 2H), 3.91 (s, 3H). ^13^C NMR (125 MHz, DMSO-D6) δ 163.61, 152.17, 142.48, 136.71, 133.59, 132.62, 129.33, 127.18, 122.67, 122.15, 119.16, 110.73, 31.80. ESI MS (*m/z*) 267.11 [M+H]^+^.

*1.1.4.3. 4-(1-Ethyl-1H-benzo[d]imidazol-2-yl)-N-hydroxybenzamide (****6b****).* 51% yield. R_f_ = 0.19 (9:1 ethyl acetate: hexanes). ^1^H NMR (500 MHz, DMSO-D_6_) δ 11.42 (s, 1H), 9.22 (s, 1H), 7.95 (d, *J* = 7.5 Hz, 2H), 7.87 (d, *J* = 8.5 Hz, 2H), 7.71 (d, *J* = 8.0 Hz, 1H), 7.67 (d, *J* = 8.0 Hz, 1H), 7.33-7.25 (m, 2H), 4.34 (q, *J* = 7.5 Hz, 2H), 1.33 (t, *J* = 7.5 Hz, 3H). ^13^C NMR (125 MHz, CD_3_OD) δ 167.1, 153.6, 143.4, 136.4, 135.2, 134.2, 130.7, 128.7, 124.6, 124.1, 119.9, 111.9, 40.8, 15.4. ESI MS (*m/z*) 282.12 [M+H]^+^.

*1.1.4.4. N-hydroxy-4-(1-propyl-1H-benzo[d]imidazol-2-yl)benzamide (****6c****).* 49% yield. R_f_ = 0.19 (9:1 ethyl acetate: hexanes). ^1^H NMR (500 MHz, DMSO-D_6_) δ 11.41 (s, 1H), 9.19 (s, 1H), 7.93 (d, *J* = 8.5 Hz, 2H), 7.86 (d, *J* = 8.5 Hz, 2H), 7.70 (d, *J* = 8.5 Hz, 2H), 7.33-7.25 (m, 2H), 4.29 (t, *J* = 7.5 Hz, 2H), 1.72-1.65 (m, 2H), 0.72 (t, *J* = 7.5 Hz, 3H). ^13^C NMR (125 MHz, DMSO-D_6_) δ 163.7, 152.1, 142.2, 135.7, 133.8, 132.9, 129.3, 127.4, 122.8, 122.3, 119.2, 111.2, 45.7, 22.7, 11.0. ESI MS (*m/z*) 296.14 [M+H]^+^.

*1.1.4.5. 4-(1-Butyl-1H-benzo[d]imidazol-2-yl)-N-hydroxybenzamide (****6d****).* 51% yield. R_f_ = 0.26 (9:1 ethyl acetate: hexanes). ^1^H NMR (500 MHz, DMSO-D_6_) δ 11.40 (s, 1H), 9.18 (s, 1H), 7.93 (d, *J* = 8.5 Hz, 2H), 7.86 (d, *J* = 8.0 Hz, 2H), 7.68 (t, *J* = 8.5 Hz, 2H), 7.32-7.24 (m, 2H), 4.32 (t, *J* = 7.0 Hz, 2H), 1.67-1.61 (m, 2H), 1.16-1.08 (m, 2H), 0.74 (t, *J* = 7.5 Hz, 3H). ^13^C NMR (125 MHz, DMSO-D_6_) δ 164.1, 152.6, 143.1, 136.2, 134.1, 133.6, 129.7, 127.8, 123.2, 122.6, 119.8, 111.6, 44.4, 31.7, 19.7, 13.8. ESI MS (*m/z*) 310.16 [M+H]^+^.

*1.1.4.6. 4-(Benzo[d]oxazol-2-yl)-N-hydroxybenzamide (****9a****).* 45% yield. R_f_ = 0.24 (7:3 ethyl acetate: hexanes). ^1^H-NMR (500 MHz, DMSO-D6) 1H-NMR (500 MHz, DMSO-D6) δ 11.45 (s, 1H), 9.21 (s, 1H), 8.27 (d, J = 8.0 Hz, 2H), 7.98 (d, J = 8.6 Hz, 2H), 7.80-7.85 (m, 2H), 7.41-7.48 (m, 2H). ^13^C NMR (125 MHz, DMSO-D6) δ 163.3, 161.5, 150.3, 141.4, 135.7, 128.6, 127.9, 127.3, 125.9, 125.1, 120.1, 111.1. ESI MS (*m/z*) 255.08 [M+H]^+^.

*1.1.4.7. 4-(Benzo[d]thiazol-2-yl)-N-hydroxybenzamide (****9b****).* 42% yield. R_f_ = 0.30 (7:3 ethyl acetate: hexanes). ^1^H NMR (500 MHz, DMSO-D_6_) δ11.41 (s, 1H), 9.19 (s, 1H), 8.19-8.17 (m, 3H), 8.10 (d, *J* =8.0 Hz, 1H), 7.94 (d, *J* = 9.0 Hz, 2H), 7.57 (t, *J* = 7.3 Hz, 1H), 7.49 (t, *J* = 7.3 Hz, 1H). ^13^C NMR (125 MHz, DMSO-D6) δ 166.4, 163.2, 153.5, 135.1, 135.0, 134.7, 127.9, 127.2, 126.9, 125.9, 123.1, 122.5. ESI MS (*m/z*) 271.05 [M+H]^+^.

*1.1.4.8. 3-(1H-benzo[d]imidazol-2-yl)-N-hydroxybenzamide (****13****).* 33% yield. ^1^H-NMR (500 MHz, CD_3_OD) δ 8.49 (s, 1H), 8.26 (d, J = 8.0 Hz, 1H), 7.85 (d, J = 7.4 Hz, 1H), 7.65 (q, J = 7.4 Hz, 3H), 7.29 (q, J = 3.1 Hz, 2H). ^13^C NMR (125 MHz, CD_3_OD) δ 167.5, 149.5, 135.4, 133.9, 133.0, 131.5, 132.8, 129.9, 127.9, 124.2, 115.1. ESI MS (*m/z*) 254.09 [M+H]^+^.

*1.1.4.9. N-hydroxy-3-(1-methyl-1H-benzo[d]imidazol-2-yl)benzamide (****14a****).* 49% yield. ^1^H-NMR (500 MHz, DMSO-D6) δ 11.45 (s, 1H), 9.24 (s, 1H), 8.23 (s, 1H), 8.01 (d, J = 8.0 Hz, 1H), 7.94 (d, J = 8.0 Hz, 1H), 7.62-7.71 (m, 3H), 7.25-7.33 (m, 2H), 3.90 (s, 3H) ^13^C NMR (125 MHz, DMSO-D6) δ 163.6, 152.4, 142.5, 136.5, 133.3, 131.9, 130.4, 129.0, 128.1, 127.7, 122.6, 122.2, 119.1, 110.8, 31.8. ESI MS (*m/z*) 268.11 [M+H]^+^.

*1.1.4.10. 3-(1-Ehyl-1H-benzo[d]imidazol-2-yl)-N-hydroxybenzamide (****14b****).* 43% yield. ^1^H NMR (500 MHz, CD_3_OD) δ 8.33 (s, 1H), 8.12 (d, *J* = 8 Hz, 1H), 8.03 (d, *J* = 8 Hz, 1H), 7.74-7.67 (m, 3H), 7.33-7.25 (m, 2H), 4.33 (q, *J* = 7 Hz, 2H), 1.36 (t, *J* = 7 Hz, 3H) ^13^C NMR (125 MHz, CD_3_OD) δ 168.4, 152,5, 141.9, 135.0, 132.9, 131.0, 130.0, 129.9, 128.9, 123.2, 122.7, 118.5, 39.4, 14.1 ESI MS (*m/z*) 282.12 [M+H]^+^.

*1.1.4.11. N-hydroxy-3-(1-propyl-1H-benzo[d]imidazol-2-yl)benzamide (****14c****).* 55% yield. R_f_ = 0.22 (8:2 ethyl acetate: hexanes). ^1^H-NMR (500 MHz, DMSO-D6) δ 11.42 (s, 1H), 9.18 (s, 1H), 8.13 (s, 1H), 7.93 (d, J = 6.9 Hz, 2H), 7.65-7.70 (m, 3H), 7.28 (td, J = 15.5, 7.6 Hz, 2H), 4.29 (t, J = 7.2 Hz, 2H), 1.68-1.73 (m, 2H), 0.74 (q, J = 7.3 Hz, 3H). ^13^C NMR (125 MHz, DMSO-D_6_) δ 163.5, 152.3, 142.6, 135.8, 133.2, 131.7, 131.2, 129.1, 128.2, 127.6, 122.6, 122.1, 119.3, 111.1, 45.6, 22.7, 11.0. ESI MS (*m/z*) 296.13 [M+H]^+^.

*1.1.4.12. 3-(1-Butyl-1H-benzo[d]imidazol-2-yl)-N-hydroxybenzamide (****14d****).* 50% yield. R_f_ = 0.28 (8:2 ethyl acetate: hexanes). ^1^H NMR (500 MHz, DMSO-D_6_) δ 11.45 (s, 1H), 9.21 (s, 1H), 8.15 (t, *J* = 1.5 Hz, 1H), 7.95-7.92 (m, 2H), 7.71-7.66 (m, 3H), 7.33-7.25 (m, 2H), 4.32 (t, *J* = 7.5 Hz, 2H), 1.69-1.63 (m, 2H), 1.18-1.10 (m, 2H), 0.74 (t, *J* = 7.5 Hz, 3H). ^13^C NMR (125 MHz, DMSO-D_6_) δ 163.4, 152.3, 142.6, 135.7, 133.3, 131.7, 130.8, 129.0, 128.0, 127.6, 122.7, 122.1, 119.3, 111.1, 43.8, 31.3, 19.2, 13.3. ESI MS (*m/z*) 310.15 [M+H]^+^.

*1.1.4.13. 3-(Bnzo[d]oxazol-2-yl)-N-hydroxybenzamide (****17a****).* 50% yield. ^1^H NMR (500 MHz, DMSO-D_6_) δ 11.52 (s, 1H), 9.24 (s, 1H), 8.61 (s, 1H), 8.33 (d, *J* = 7.5 Hz, 1H), 8.0 (d, *J* = 8.0 Hz, 1H), 7.83 (t, *J* = 8.0 Hz, 2H), 7.71 (t, *J* = 8.0 Hz, 1H), 7.46-7.43 (m, 2H). ^13^C NMR (125 MHz, DMSO-D_6_) δ 163.2, 161.7, 150.3, 141.4, 133.8, 130.1, 129.4, 129.7, 126.7, 125.9, 125.1, 120.0, 111.1. ESI MS (*m/z*) 255.08 [M+H]^+^.

*1.1.4.14. 3-(Benzo[d]thiazol-2-yl)-N-hydroxybenzamide (****17b****).* 34% yield. ^1^H-NMR (500 MHz, DMSO-D6) δ 11.50 (s, 1H), 9.21 (s, 1H), 8.47 (s, 1H), 8.22 (dd, J = 22.1, 7.7 Hz, 2H), 8.11 (d, J = 8.0 Hz, 1H), 7.94 (d, J = 8.0 Hz, 1H), 7.67 (t, J = 7.7 Hz, 1H), 7.58 (t, J = 7.2 Hz, 1H), 7.50 (t, J = 7.4 Hz, 1H). ^13^C NMR (125 MHz, DMSO-D_6_) δ 166.6, 163.3, 153.5, 134.6, 133.9, 133.1, 129.8, 129.7, 129.6, 126.9, 125.8, 125.6, 123.1, 122.6. ESI MS (*m/z*) 271.05 [M+H]^+^.

*1.1.5. Methyl 4-(benzo[d]oxazol-2-yl)benzoate (****8a****)*

2-Aminophenol (0.20 g, 1.83 mmol) and methyl 4-formylbenzoate (0.30 g, 1.83 mmol) were dissolved in dichloromethane (20 ml) and was stirred at rt for 4 h under argon atmosphere. Iodine (0.12 g, 0.92 mmol) was added to the reaction mixture and the reaction mixture was stirred at room temperature for 1 h under open flask. The mixture was concentrated under reduced pressure and purified by column to afford compound **8a** in 32% yield. R_f_ = 0.31 (1:9 ethyl acetate: hexanes). ^1^H NMR (500 MHz, CDCl_3_) δ 8.32 (d, *J* = 8.5 Hz, 2H), 8.18 (d, *J* = 8.5 Hz, 2H), 7.80-7.79 (m, 1H), 7.61-7.59 (m, 1H), 7.40-7.37 (m, 2H), 3.96 (s, 3H). ^13^C NMR (125 MHz, CDCl_3_) δ 166.5, 162.1, 151.0, 142.1, 132.6, 131.1, 130.2, 127.6, 125.9, 125.0, 120.5, 110.9, 52.6.

*1.1.6. Methyl 4-(benzo[d]thiazol-2-yl)benzoate (****8b****)*

2-Aminothiophenol (1.71 mL, 15.98 mmol) and methyl 4-formylbenzoate (2.62 g, 15.98 mmol) were dissolved in dichloromethane (100 ml) and was stirred at rt for 4 h under argon atmosphere. Iodine (1.01 g, 7.99 mmol) was added to the reaction mixture and the reaction mixture was stirred at room temperature for 45 min under open flask. The mixture was concentrated under reduced pressure and purified by column to afford compound **8b** in 35% yield. R_f_ = 0.31 (1:9 ethyl acetate: hexanes). ^1^H NMR (500 MHz, CDCl_3_) δ 8.15-8.14 (m, 4H), 8.09 (d, *J* = 7.5 Hz, 1H), 7.91 (d, *J* = 8.5 Hz, 1H), 7.51 (t, *J* = 8.0 Hz, 1H), 7.41 (t, *J* = 7.5 Hz, 1H), 3.95 (s, 3H). ^13^C NMR (125 MHz, CDCl_3_) δ 166.5, 154.2, 137.6, 135.4, 132.2, 130.4, 129.7, 127.6, 126.7, 125.8, 123.7, 121.9, 52.5.

*1.1.7. Methyl 3-(1H-benzo[d]imidazol-2-yl)benzoate (****11****)*

A mixture of *o*-phenylenediamine (2.00 g, 18.50 mmol) and methyl 3-formylbenzoate (3.03 g, 18.50 mmol) in DMF (180 mL) and water (20 mL) was stirred at 80 ^o^C for 48 h under open flask. The mixture was concentrated under reduced pressure and purified by column to afford compound **11** in 86% yield. R_f_ = 0.25 (3:7 ethyl acetate: hexanes). ^1^H NMR (500 MHz, CDCl_3_) δ 8.66 (d, J = 1.7 Hz, 1H), 8.37-8.39 (m, 1H), 8.08-8.10 (m, 1H), 7.68 (q, J = 2.9 Hz, 2H), 7.54 (t, J = 7.7 Hz, 1H), 7.30 (q, J = 3.1 Hz, 2H), 3.89 (s, 3H). ^13^C NMR (125 MHz, CDCl_3_) δ 166.57, 150.81, 131.37, 131.16, 131.00, 130.23, 129.41, 127.58, 123.38, 52.44. ESI MS (*m/z*) 253.10 [M+H]^+^.

*1.1.8. Methyl 3-(benzo[d]oxazol-2-yl)benzoate (****16a****)*

2-Aminophenol (0.50 g, 4.58 mmol) and methyl 3-formylbenzoate (0.75 g, 4.58 mmol) in DMF (40 ml) were added in molecular sieve. Then, the reaction mixture was stirred at 60 ^o^C for 4 h under open flask. After 4 h, the reaction mixture was cooled to room temperature and NaCN (0.02 g, 0.46 mmol) was added to the reaction mixture. The reaction mixture was stirred at rt for 24 h. The mixture was concentrated under reduced pressure and purified by MPLC to afford compound **16a** in 67% yield. R_f_ = 0.25 (1:9 ethyl acetate: hexanes). ^1^H NMR (500 MHz, DMSO-D_6_) δ 8.70 (s, 1H), 8.43 (d, *J* = 8.5 Hz, 1H), 8.17 (d, *J* = 8.0 Hz, 1H), 7.84-7.82 (m, 2H), 7.77 (t, *J* = 7.5 Hz 1H), 7.48-7.41 (m, 2H), 3.92 (s, 1H). ^13^C NMR (125 MHz, CDCl_3_) δ 166.3, 162.2, 150.9, 142.1, 132.5, 131.8, 131.2, 129.3, 128.8, 127.7, 125.6, 124.9, 120.3, 52.5. ESI MS (*m/z*) 254.08 [M+H]^+^.

*1.1.9. Mthyl 3-(benzo[d]thiazol-2-yl)benzoate (****16b****)*

2-Aminothiophenol (0.50 g, 4.58 mmol) and methyl 3-formylbenzoate (0.66 g, 4.58 mmol) in DMF (40 ml) were added in molecular sieve. Then, the reaction mixture was stirred at 60 ^o^C for 4 h under open flask. After 4 h, the reaction mixture was cooled to rt and NaCN (0.02 g, 0.46 mmol) was added to the reaction mixture. The reaction mixture was stirred at rt for 24 h. The mixture was concentrated under reduced pressure and purified by MPLC to afford compound **16b** in 44% yield. R_f_ = 0.26 (1:9 ethyl acetate: hexanes). ^1^H NMR (500 MHz, DMSO-D_6_) δ 8.57 (s, 1H), 8.26 (d, *J* = 7.5 Hz, 1H), 8.13 (d, *J* = 7.5 HZ, 1H), 8.08 (d, *J* = 8.5 Hz, 2H), 7.68 (t, *J* = 7.5 Hz, 1H), 7.55 (t, *J* = 8.0 Hz, 1H), 7.46 (t, *J* = 8.0 Hz, 1H), 3.90 (s, 3H). ^13^C NMR (125 MHz, CDCl_3_) δ 166.8, 166.4, 154.1, 135.1, 134.0, 131.8, 131.6, 131.1, 129.2, 128.6, 126.5, 126.5, 125.5, 123.4, 52.4. ESI MS (*m/z*) 270.06 [M+H]^+^.


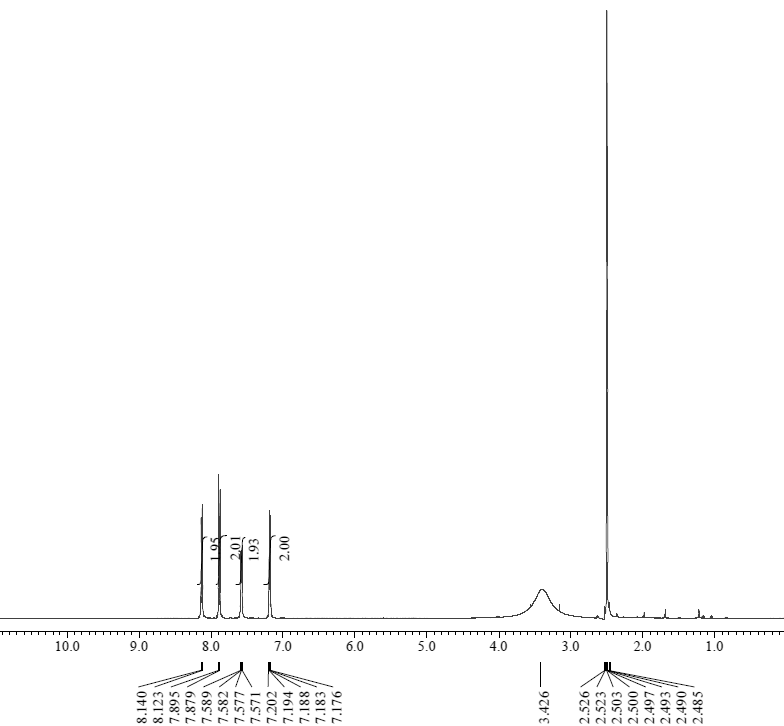


^1^H spectrum of compound 5 (500 MHz, (CD_3_)_2_SO)


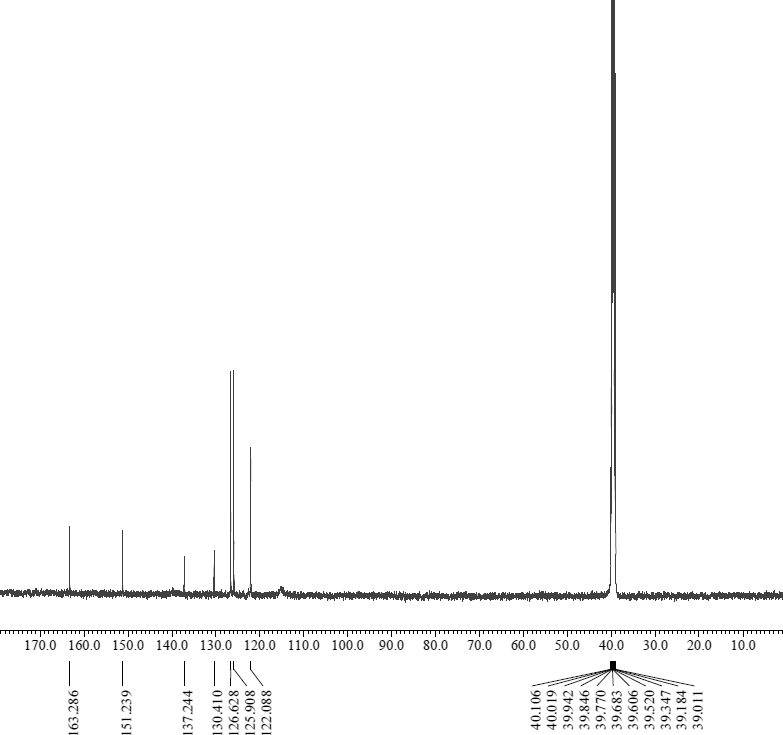


^13^C spectrum of compound 5 (125 MHz, (CD_3_)_2_SO)


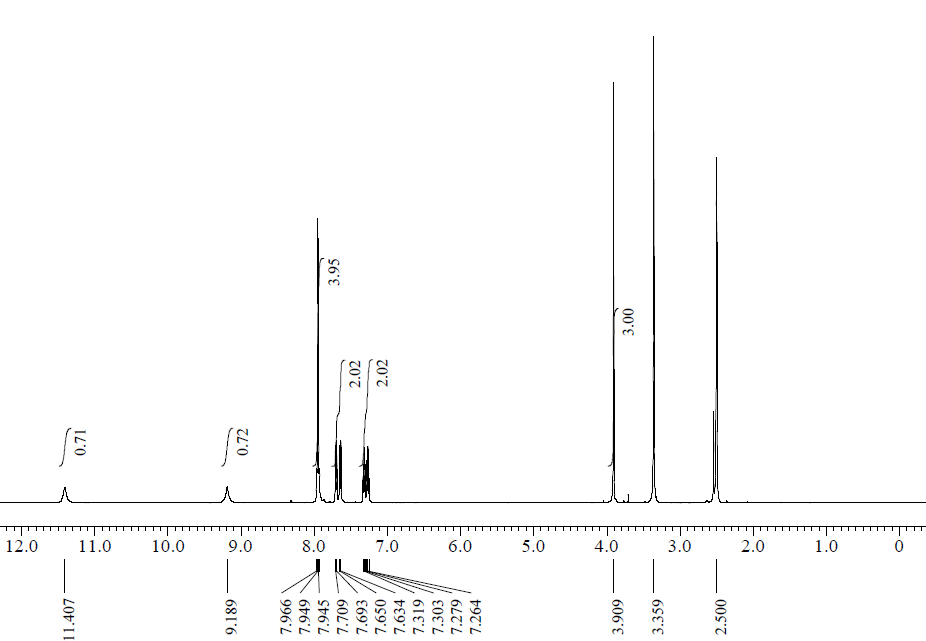


^1^H spectrum of compound 6a (500 MHz, (CD_3_)_2_SO)


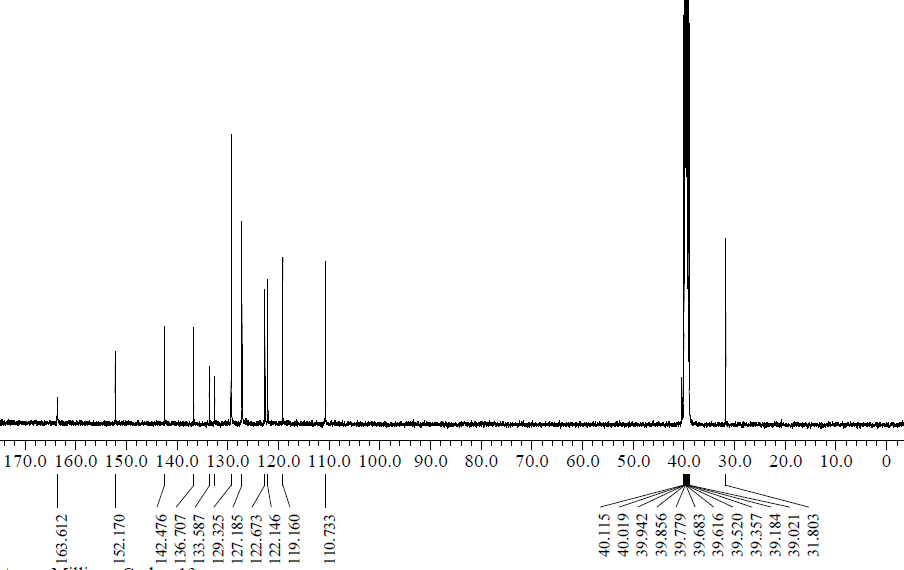


^13^C spectrum of compound 6a (125 MHz, (CD_3_)_2_SO)


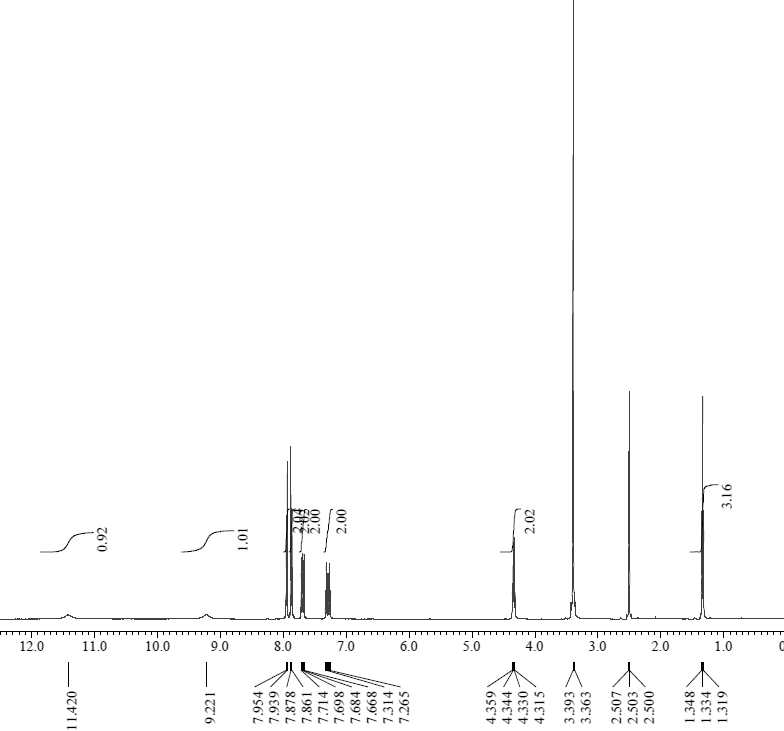


^1^H spectrum of compound 6b (500 MHz, (CD_3_)_2_SO)


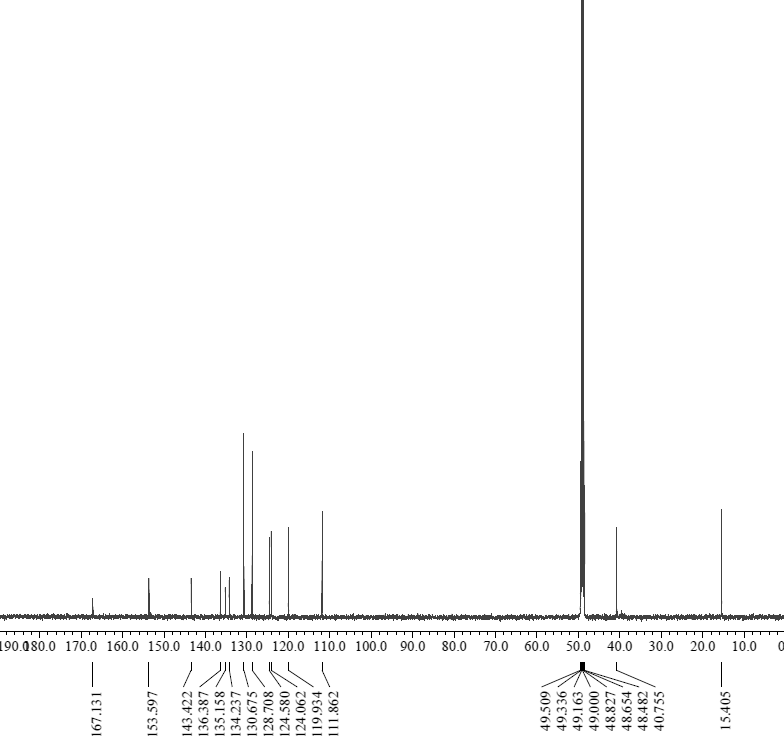


^13^C spectrum of compound 6b (125 MHz, CD_3_OD)


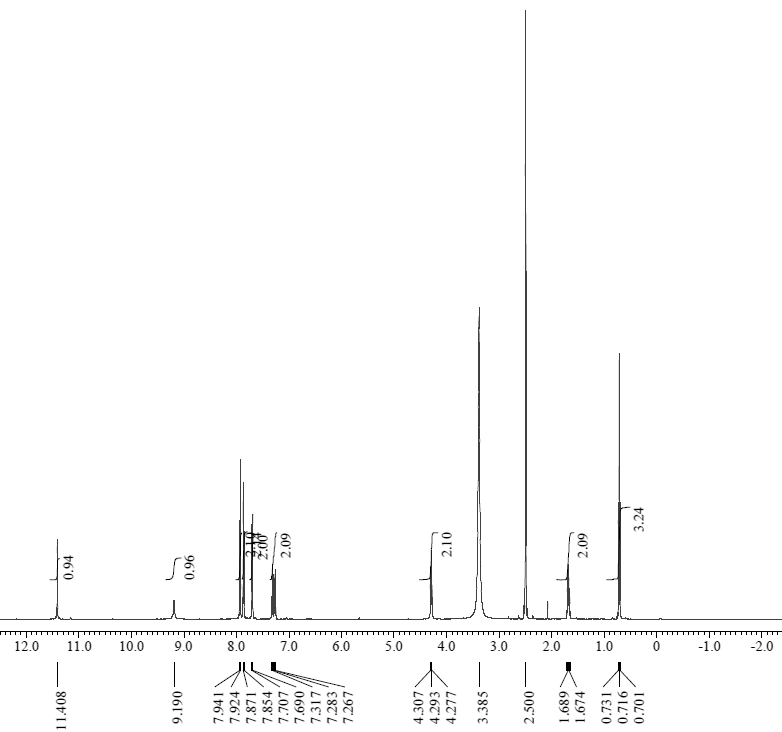


^1^H spectrum of compound 6c (500 MHz, (CD_3_)_2_SO)


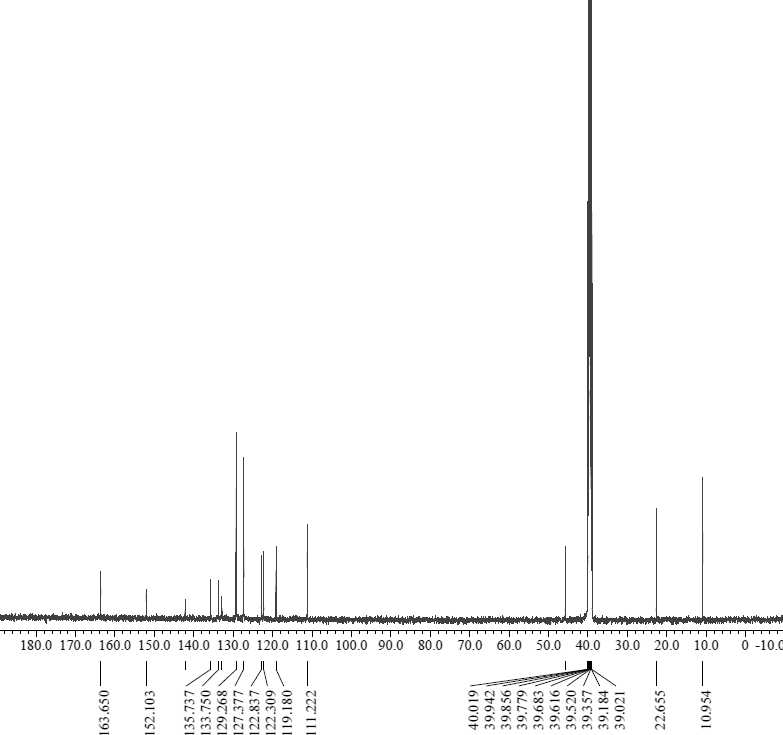


^13^C spectrum of compound 6c (125 MHz, (CD_3_)_2_SO)


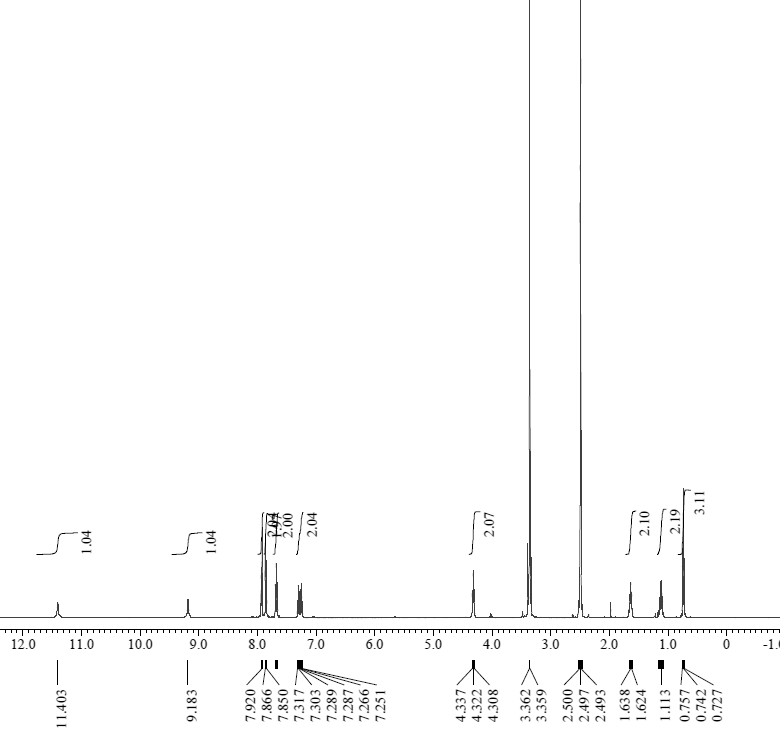


^1^H spectrum of compound 6d (500 MHz, (CD_3_)_2_SO)


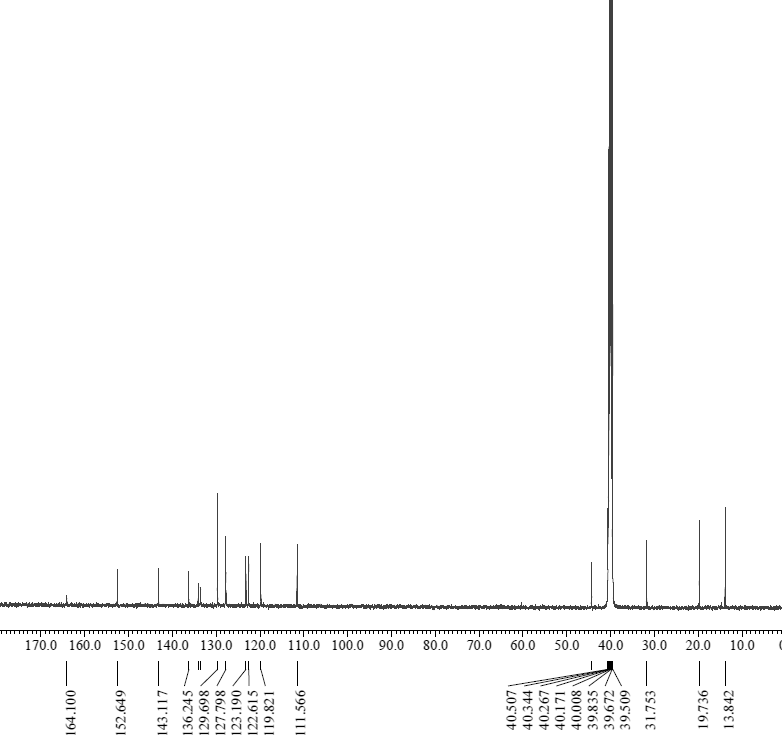


^13^C spectrum of compound 6d (125 MHz, (CD_3_)_2_SO)


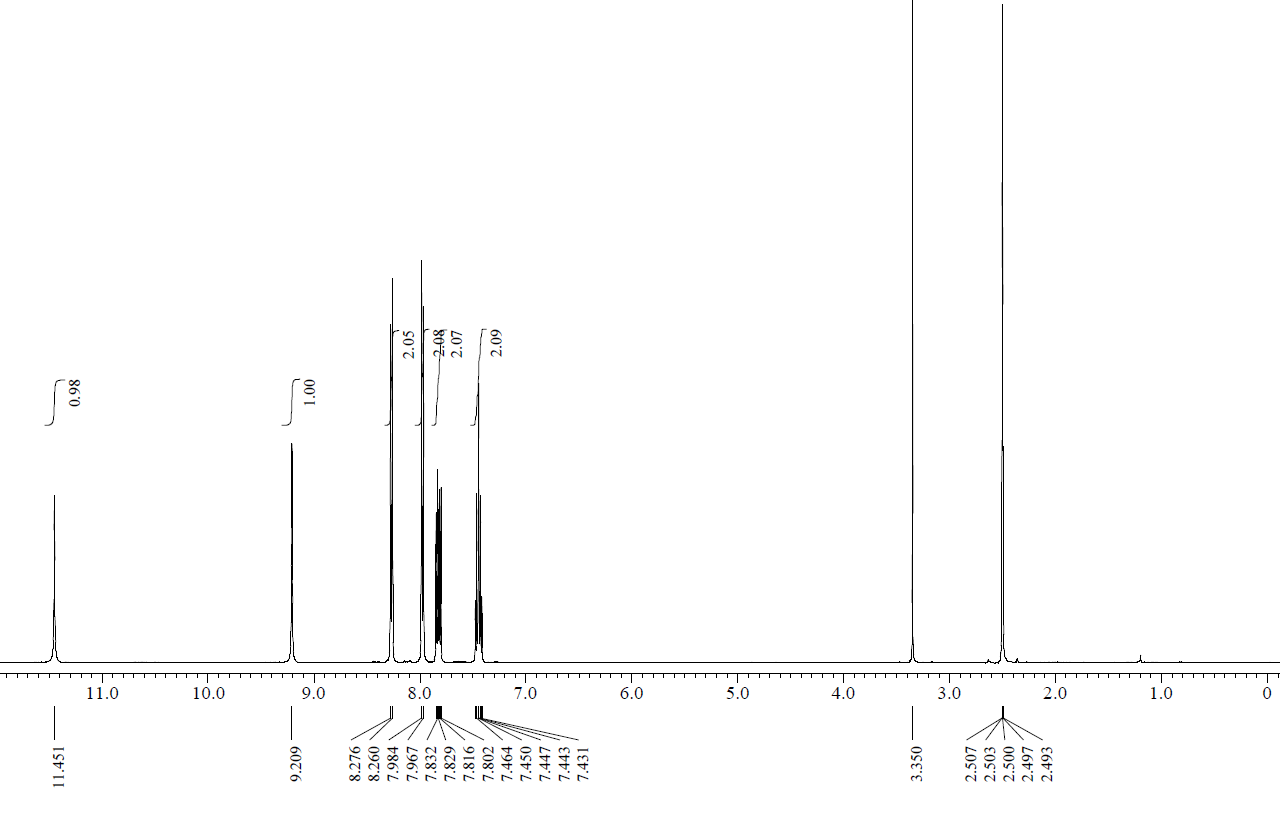


^1^H spectrum of compound 9a (500 MHz, (CD_3_)_2_SO)


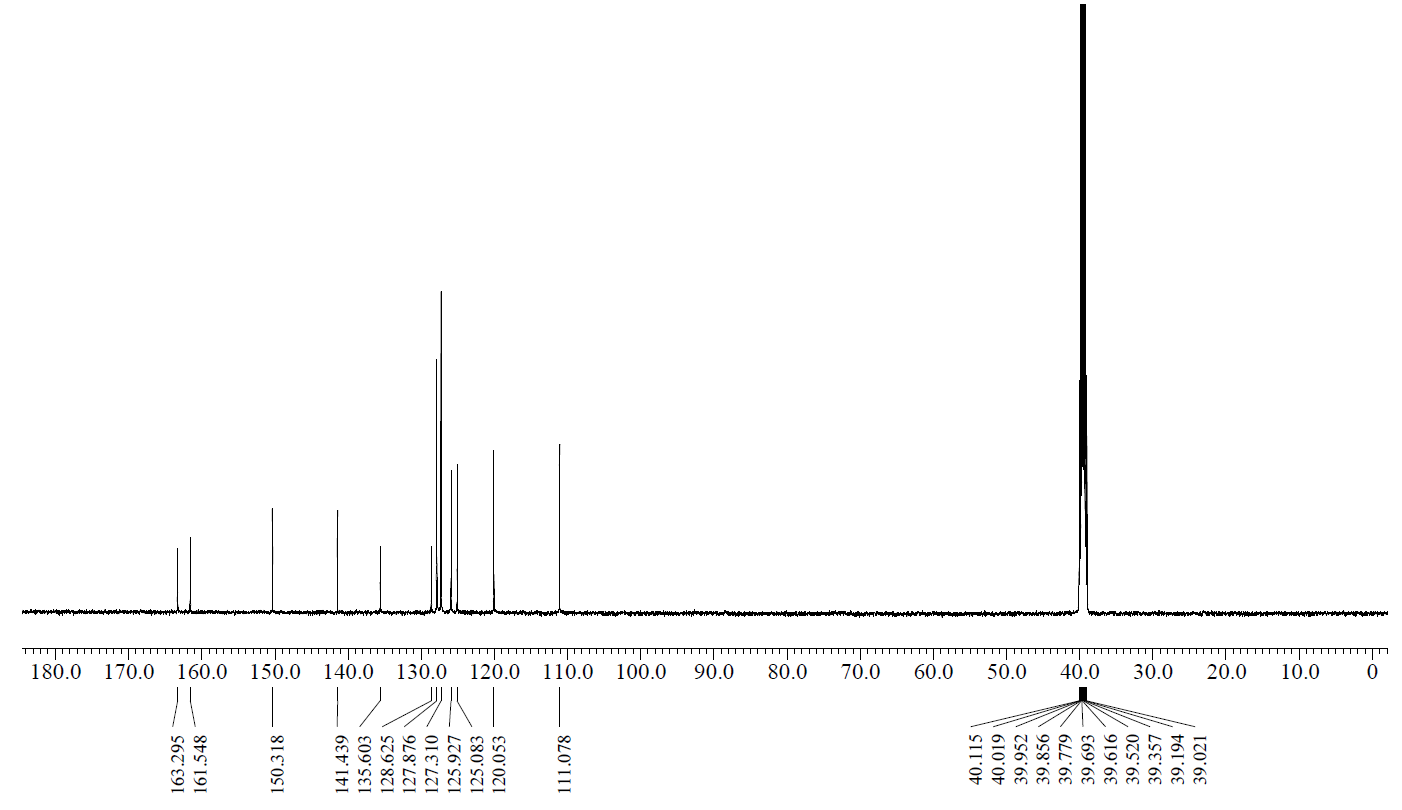


^13^C spectrum of compound 9a (125 MHz, (CD_3_)_2_SO)


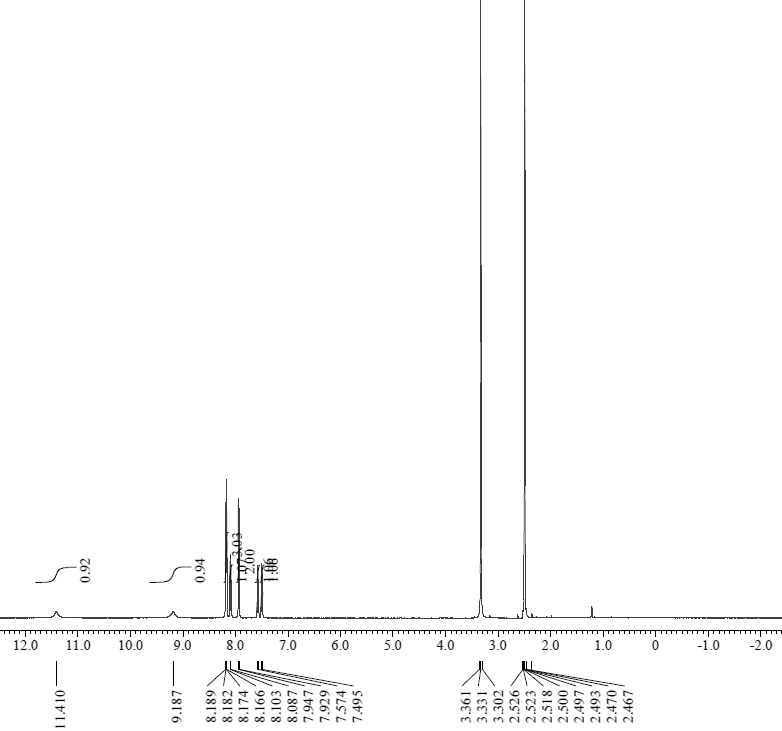


^1^H spectrum of compound 9b (500 MHz, (CD_3_)_2_SO)


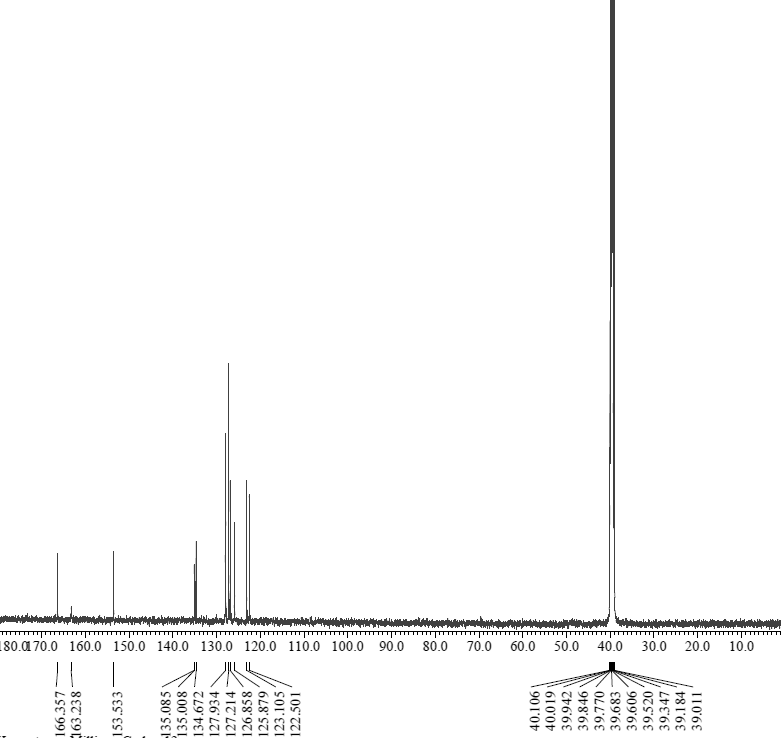


^13^C spectrum of compound 9b (125 MHz, (CD_3_)_2_SO)


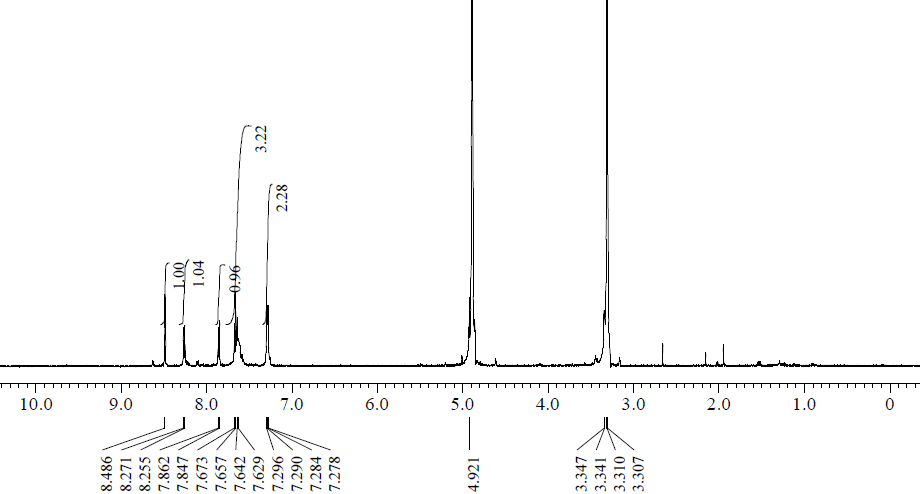


^1^H spectrum of compound 13 (500 MHz, (CD_3_OD)


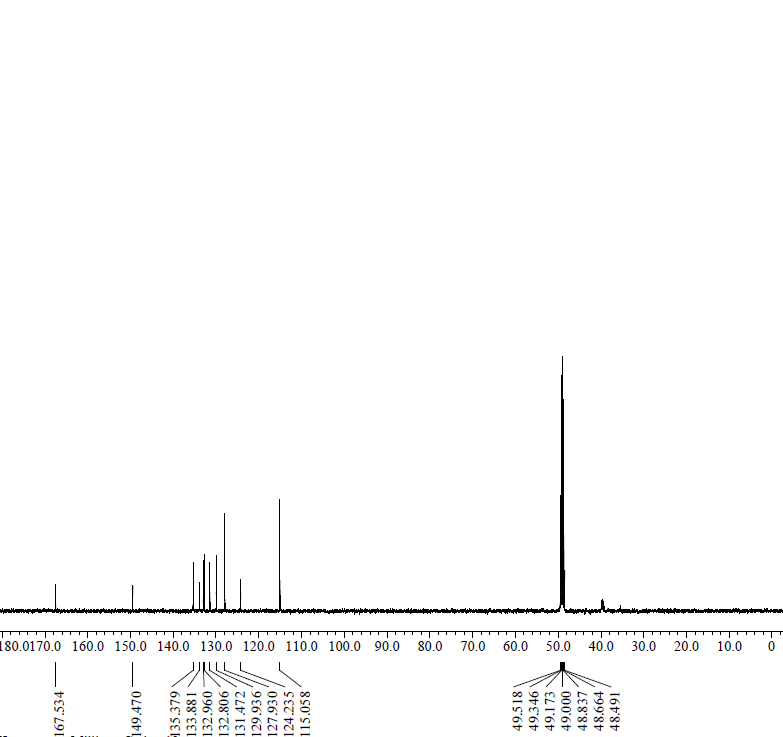


^13^C spectrum of compound 13 (125 MHz, CD_3_OD)


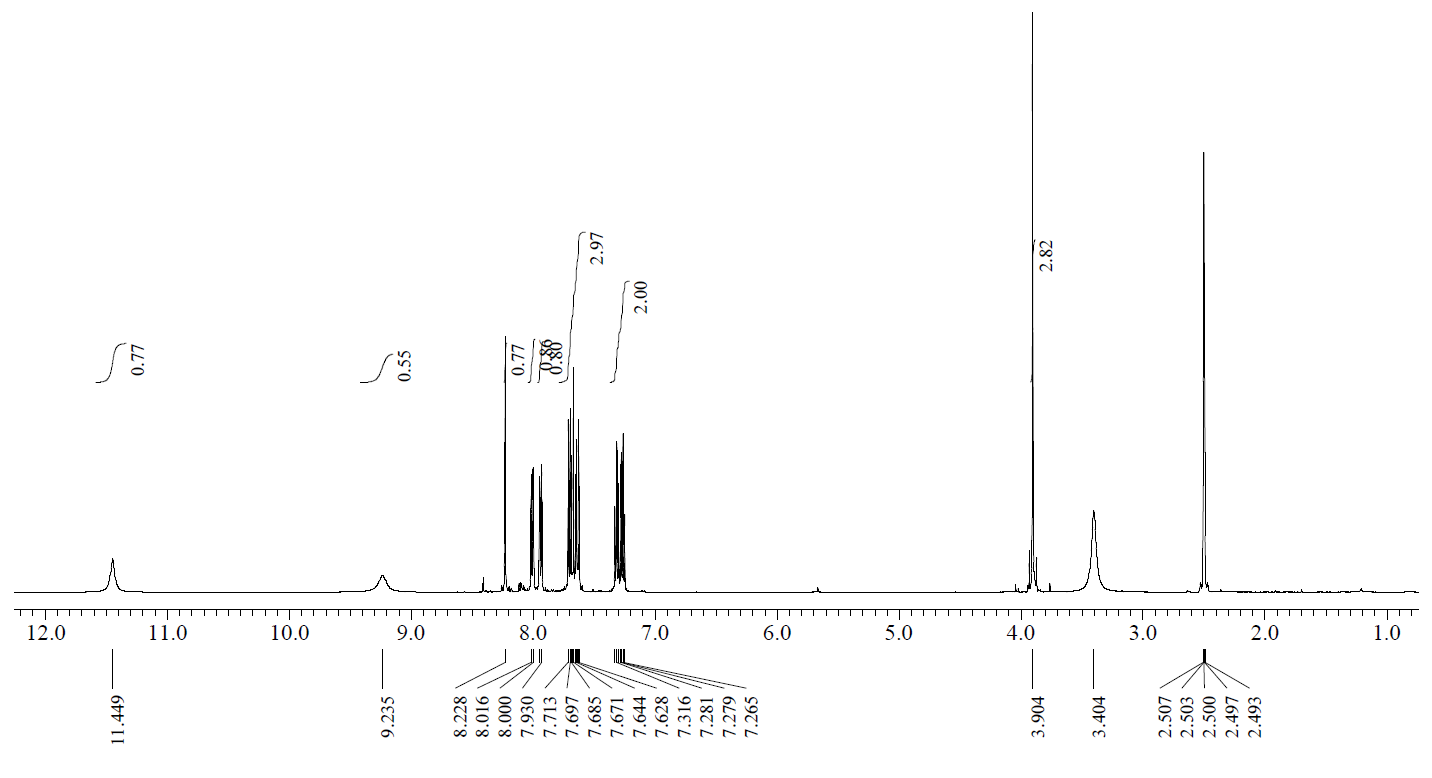


^1^H spectrum of compound 14a (500 MHz, (CD_3_)_2_SO)


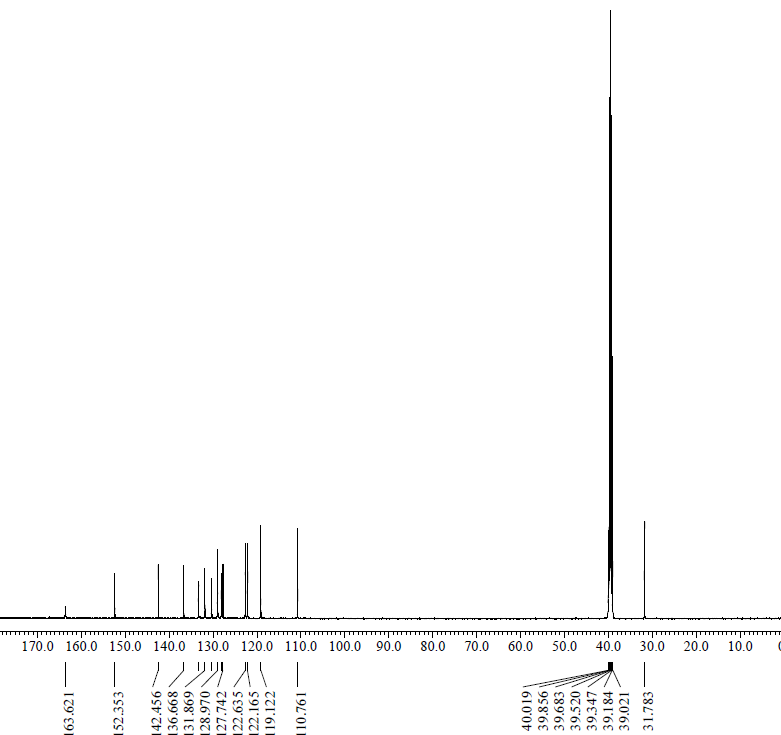


^13^C spectrum of compound 14a (125 MHz, (CD_3_)_2_SO)


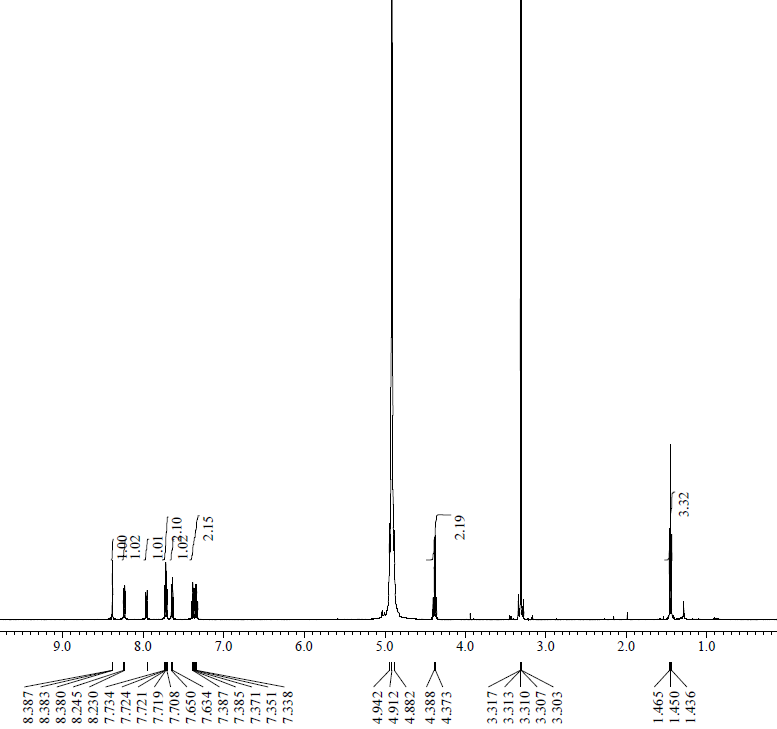


^1^H spectrum of compound 14b (500 MHz, CD_3_OD)


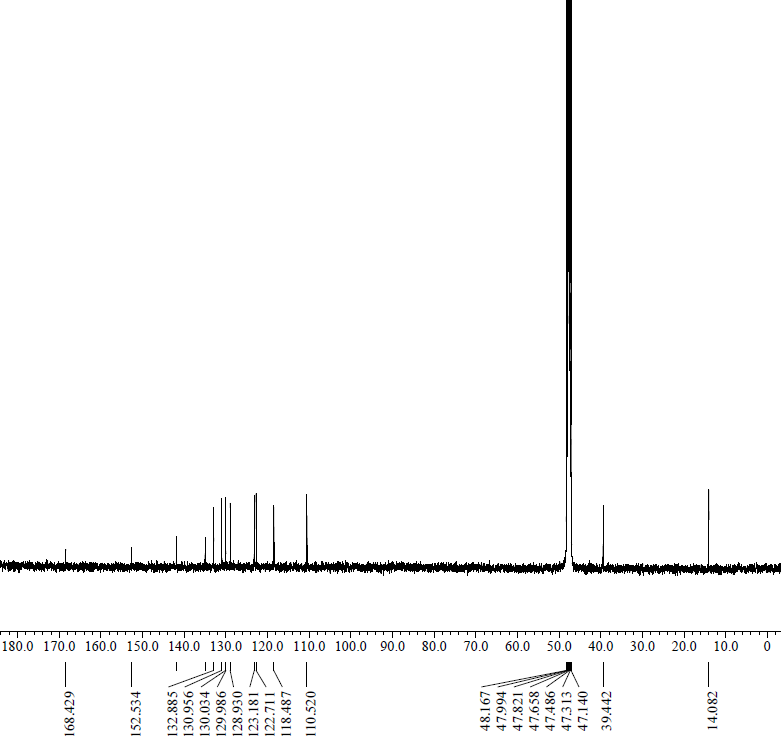


^13^C spectrum of compound 14b (125 MHz, CD_3_OD)


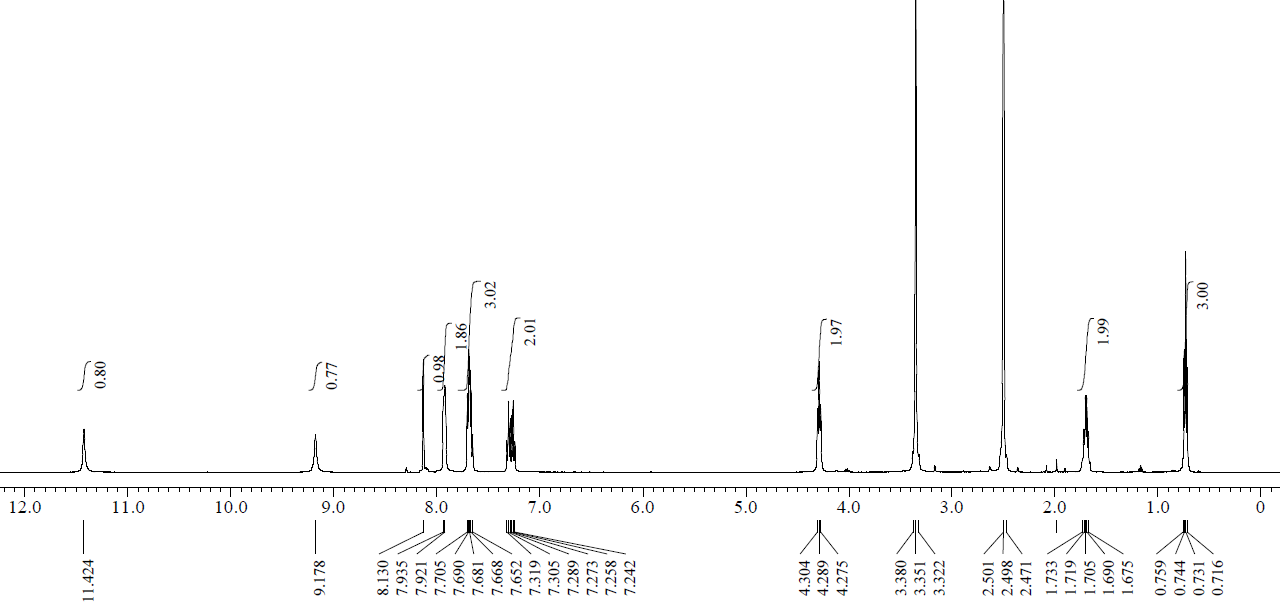


^1^H spectrum of compound 14c (500 MHz, (CD_3_)_2_SO)


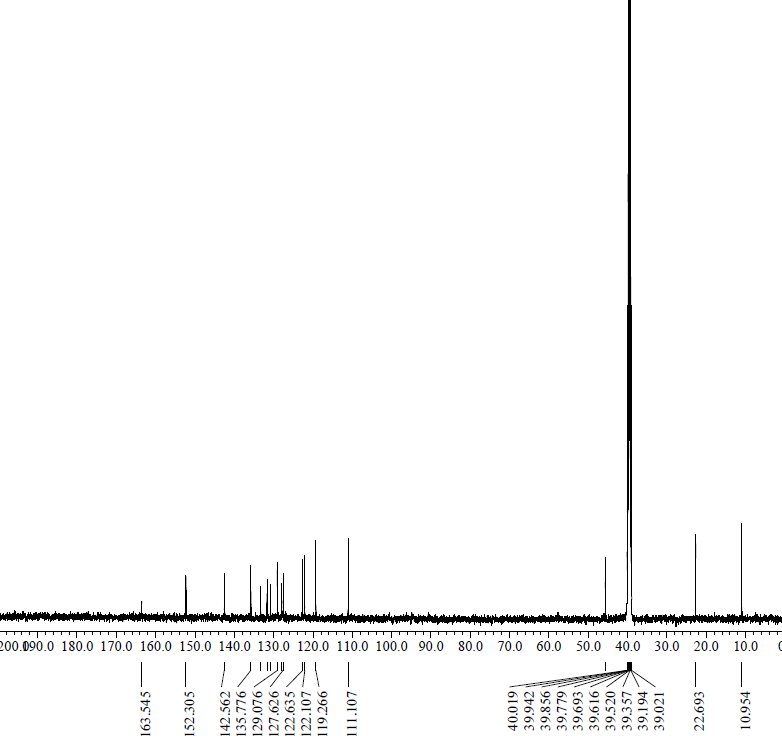


^13^C spectrum of compound 14c (125 MHz, (CD_3_)_2_SO)


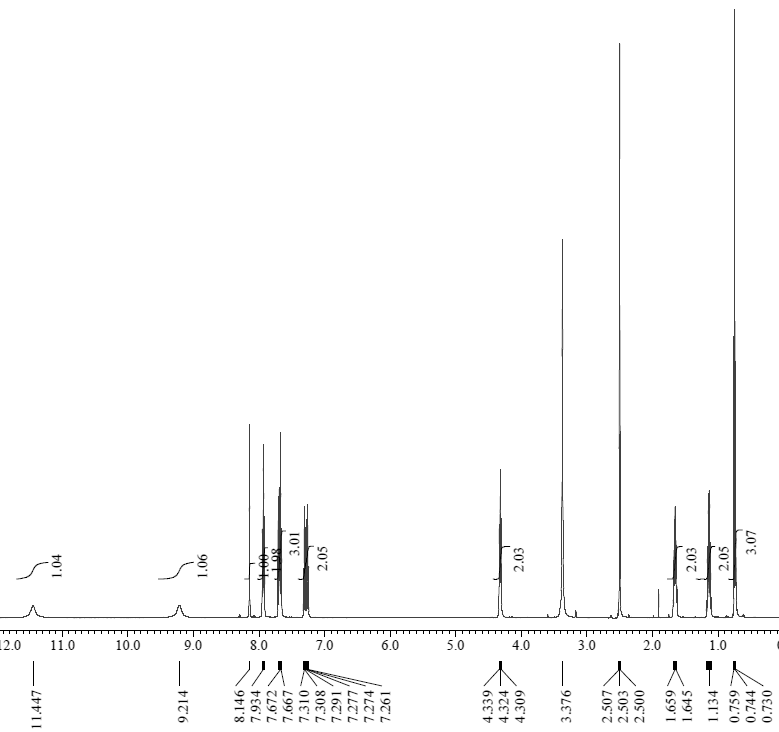


^1^H spectrum of compound 14d (500 MHz, (CD_3_)_2_SO)


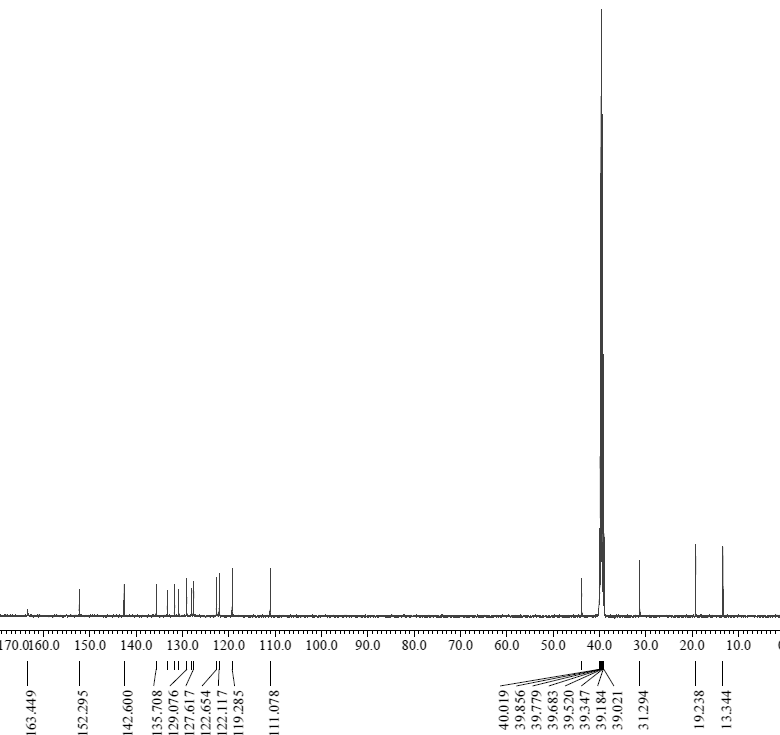


^13^C spectrum of compound 14d (125 MHz, (CD_3_)_2_SO)


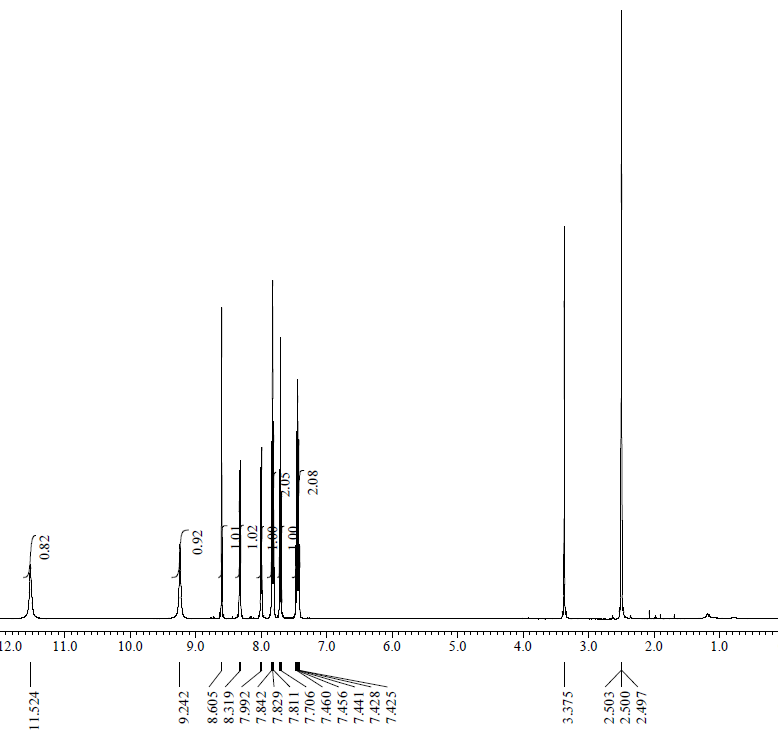


^1^H spectrum of compound 17a (500 MHz, (CD_3_)_2_SO)


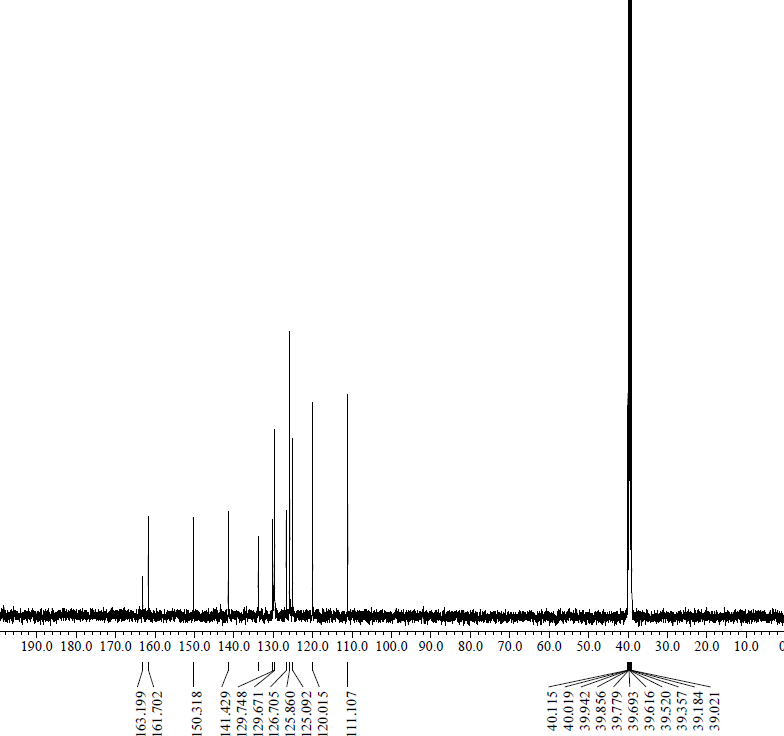


^13^C spectrum of compound 17a (125 MHz, (CD_3_)_2_SO)


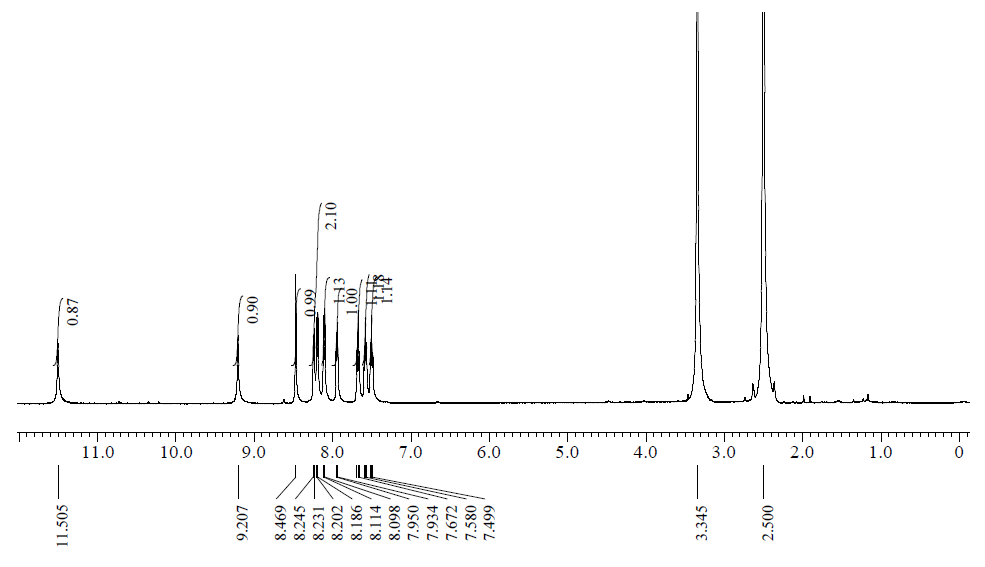


^1^H spectrum of compound 17b (500 MHz, (CD_3_)_2_SO)


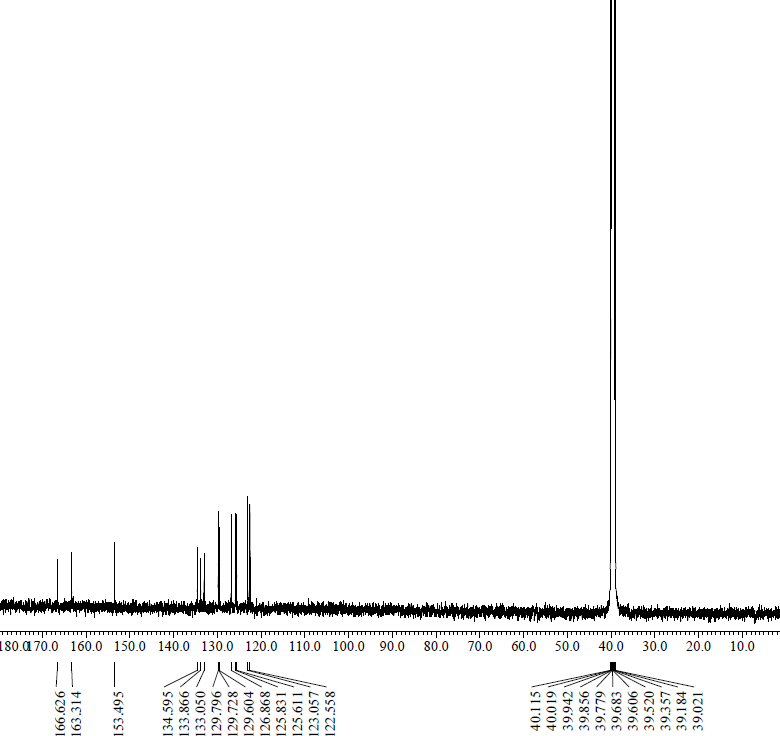


^13^C spectrum of compound **17b** (125 MHz, (CD_3_)_2_SO)


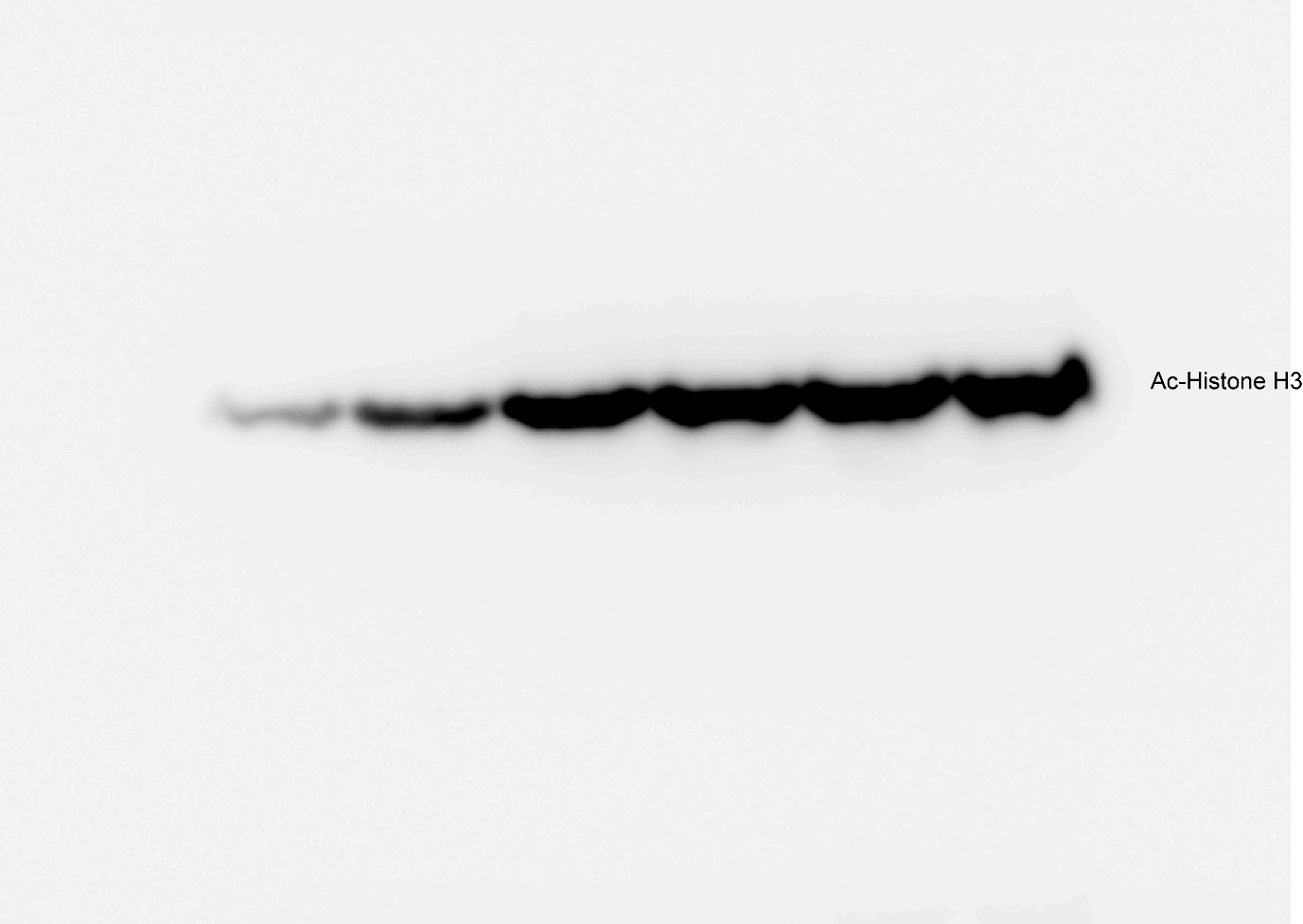


**Supplementary Figure 2**. Full-length blots for the protein expression of Ac-Histone H3. Blot was cut in two before being incubated with specific antibody and used for Figure 5.


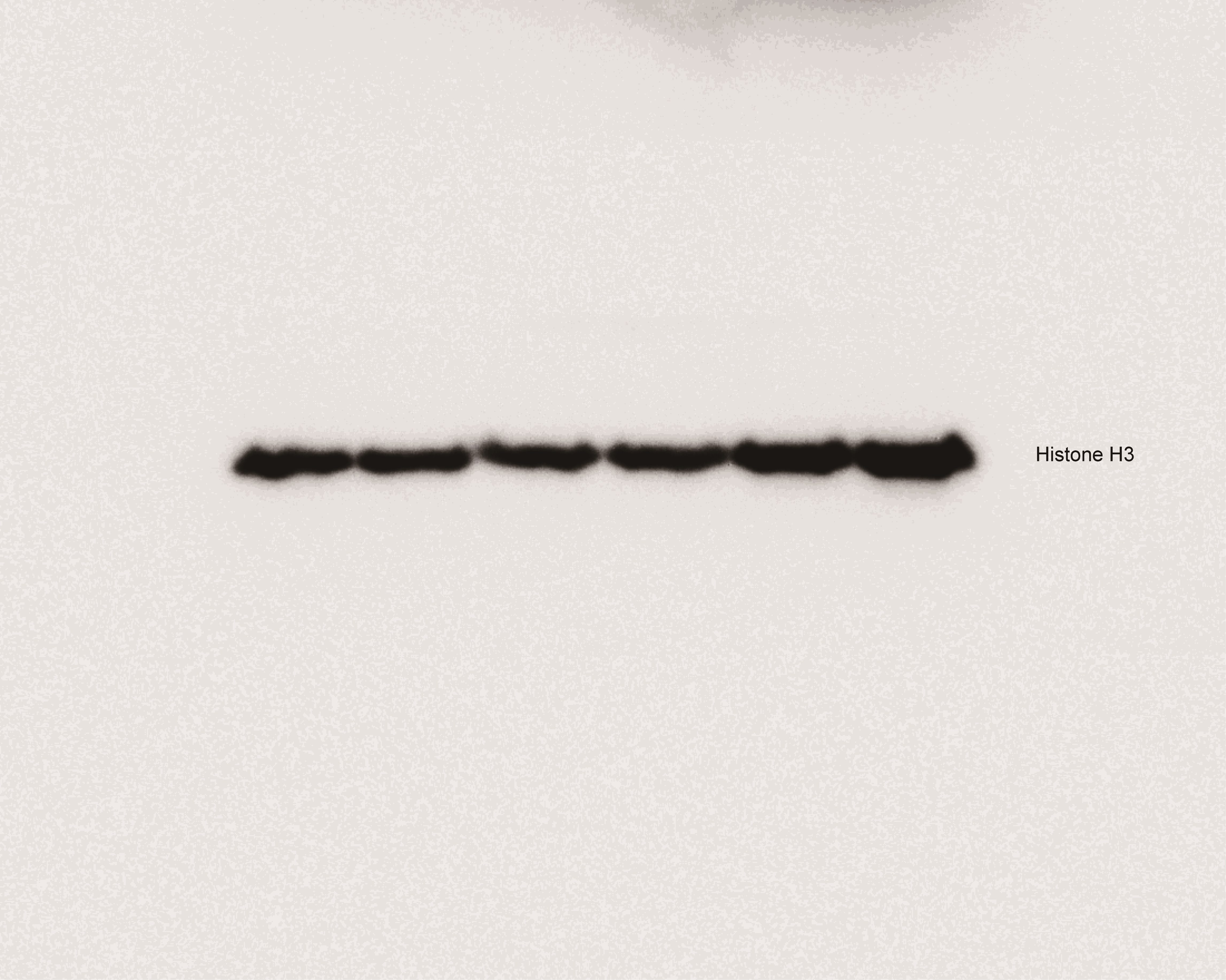


**Supplementary Figure 3**. Full-length blots for the protein expression of Histone H3. Blot was cut in two before being incubated with specific antibody and used for Figure 5.


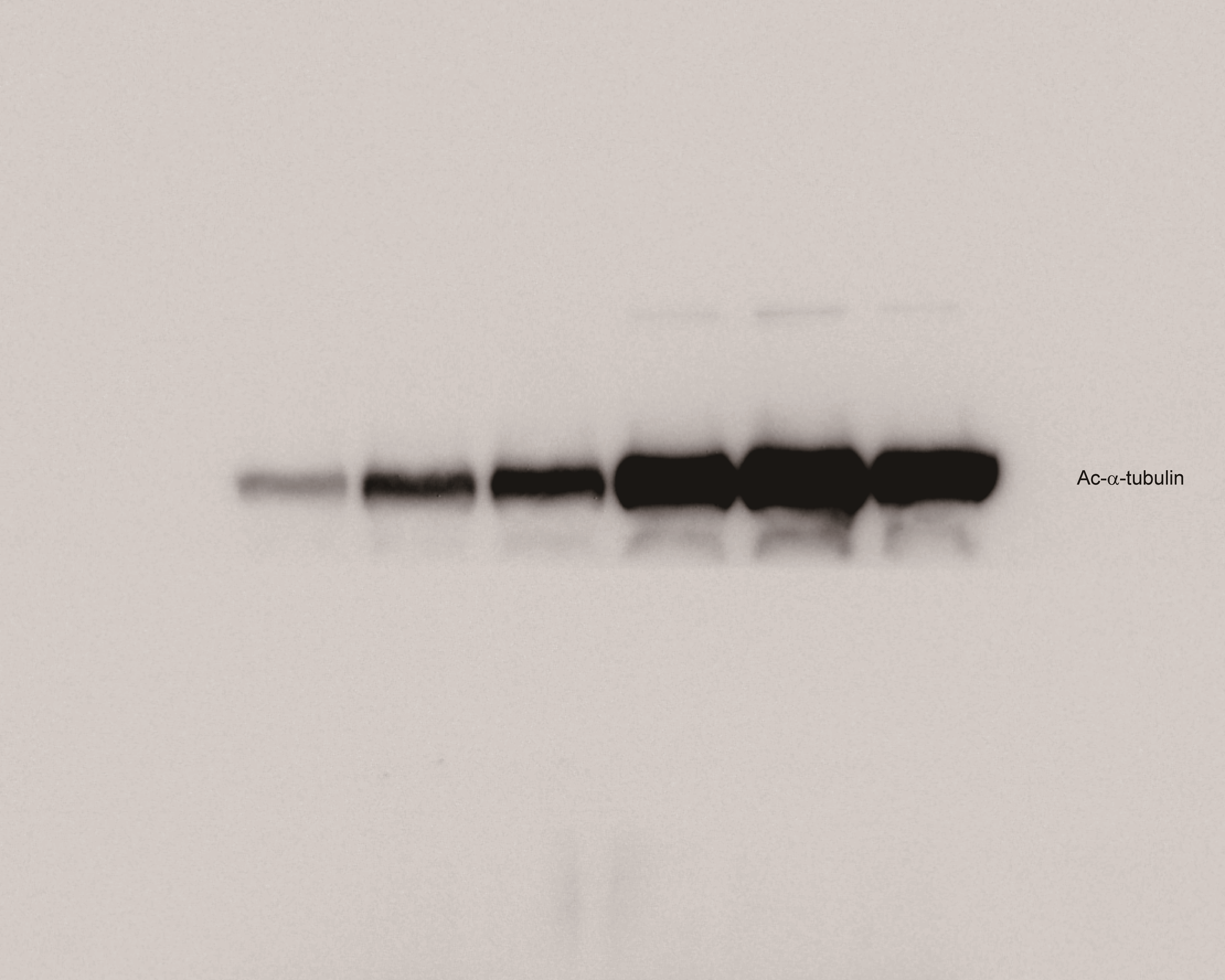


**Supplementary Figure 4**. Full-length blots for the protein expression of Ac-*α*-tubulin. Blot was cut in two before being incubated with specific antibody and used for Figure 5.


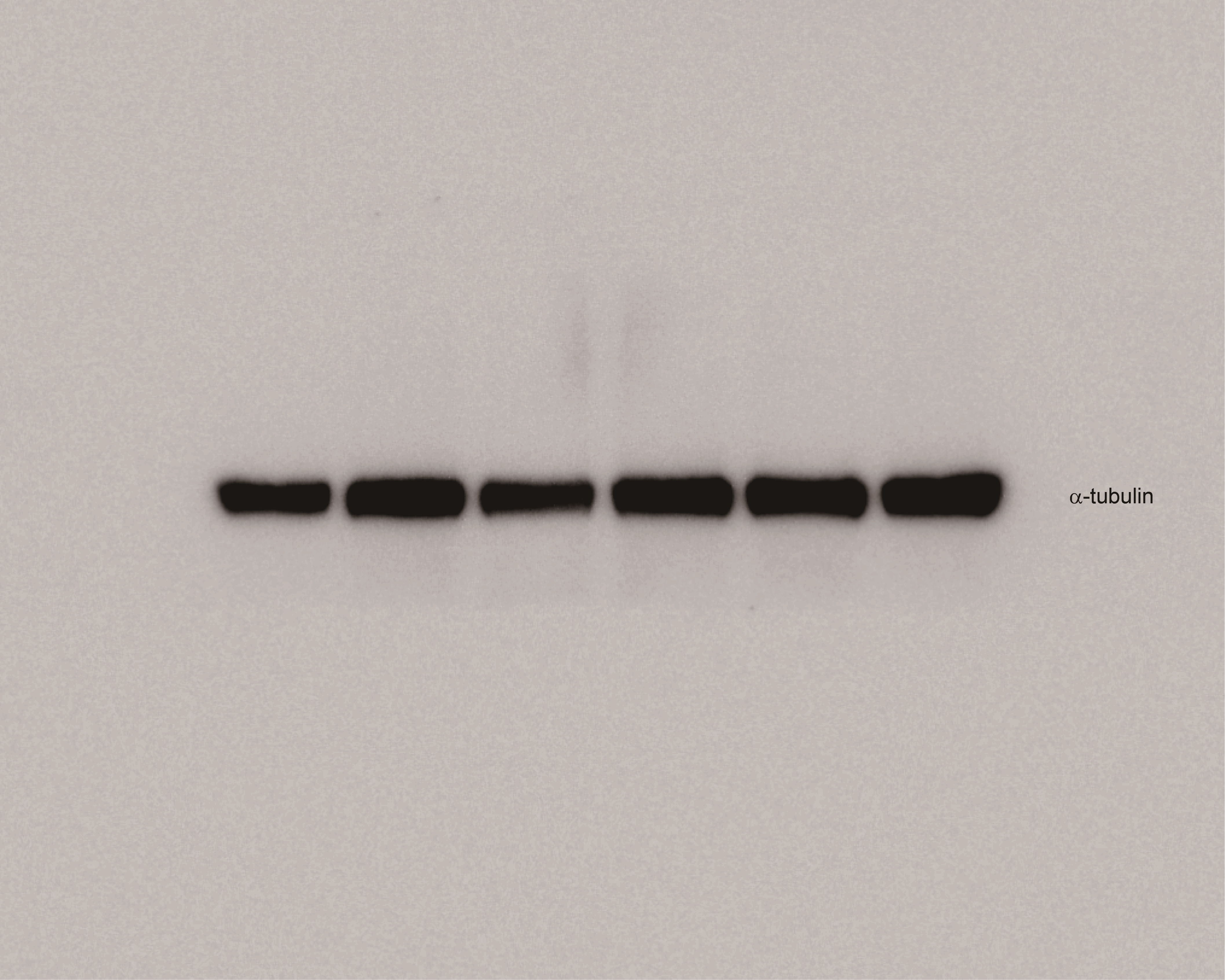


**Supplementary Figure 5**. Full-length blots for the protein expression of *α*-tubulin. Blot was cut in two before being incubated with specific antibody and used for Figure 5.


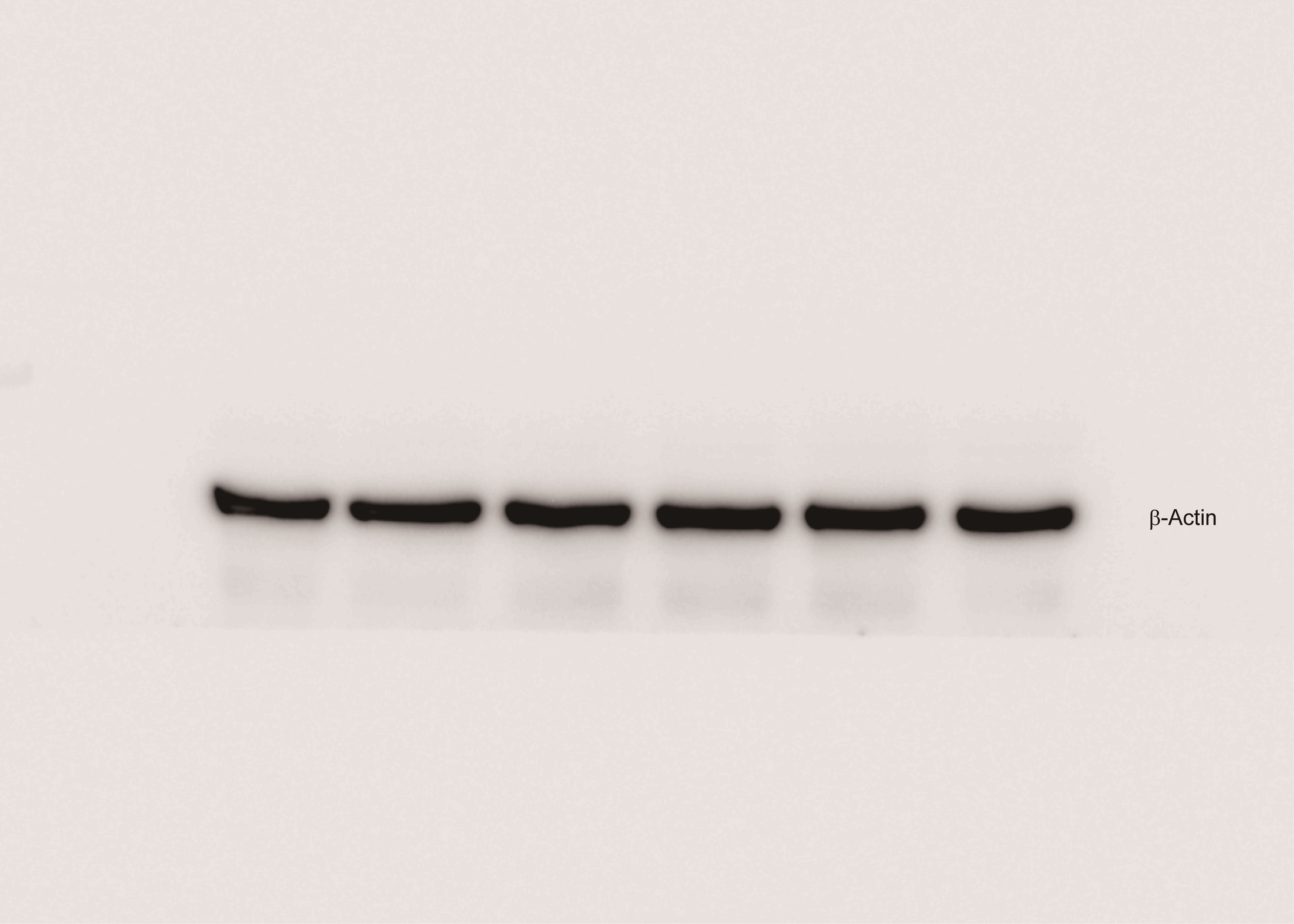


**Supplementary Figure 6**. Full-length blots for the protein expression of *β*-actin. Blot was cut in two before being incubated with specific antibody and used for Figure 5.
